# Extraction Strategy and C18 Solid-Phase Fractionation Shape Phenolic Profiles, Antioxidant Capacity, and Cancer Cell Antiproliferative Activity of Selected Medicinal Plants

**DOI:** 10.3390/antiox15070870

**Published:** 2026-07-13

**Authors:** Domantas Armonavičius, Audrius Maruška, Kristina Bimbiraitė-Survilienė, Mantas Stankevičius, Baltramiejus Jakštys, Tomas Drevinskas, Ugnė Gabrytė, Elżbieta Skrzydlewska, Ona Ragažinskienė, Vilma Kaškonienė, Saulius Šatkauskas, Inga Pečiulienė, Arvydas Kanopka

**Affiliations:** 1 Instrumental Analysis Open Access Centre, Faculty of Natural Sciences, Vytautas Magnus University, Universiteto St. 10, LT-53361 Akademija, Kauno Rajonas, Lithuania; domantas.armonavicius@vdu.lt (D.A.); kristina.bimbiraite-surviliene@vdu.lt (K.B.-S.); mantas.stankevicius@vdu.lt (M.S.); tomas.drevinskas@gmail.com (T.D.); ugne.gabryte@stud.vdu.lt (U.G.); vilma.kaskoniene@vdu.lt (V.K.); inga.peciuliene@bti.vu.lt (I.P.); arvydas.kanopka@vdu.lt (A.K.); 2 Research on Delivery of Medicine and Genes Cluster, Faculty of Natural Sciences, Vytautas Magnus University, LT-44001 Akademija, Kauno Rajonas, Lithuania; baltramiejus.jakstys@vdu.lt (B.J.); saulius.satkauskas@vdu.lt (S.Š.); 3 Department of Analytical Chemistry, Medical University of Białystok, 15-222 Białystok, Poland; elzbieta.skrydlewska@umb.edu.pl; 4 Botanical Garden, Vytautas Magnus University, LT-46324 Kaunas, Lithuania; ona.ragazinskiene@vdu.lt; 5 Department of Biological DNA Modification, Institute of Biotechnology, Life Sciences Center, Vilnius University, LT-10257 Vilnius, Lithuania

**Keywords:** medicinal plants, phenolic compounds, phenolic profiling, vegetation stage, solid-phase extraction, antiproliferative activity, antioxidant activity

## Abstract

Medicinal plants are a rich source of biologically active compounds, including phenolic acids, flavonoids, ellagitannins and other secondary metabolites. However, the contribution of specific groups of phenolic compounds to antiproliferative activity remains insufficiently clarified. This study extends our previous crude-extract screening by evaluating whether C18 solid-phase extraction (SPE) fractions with different phenolic profiles are associated with different antiproliferative responses. In parallel, extraction strategies were compared to assess method-dependent changes in phenolic recovery and antioxidant capacity, and an additional single-species vegetation-stage analysis of *Chamaenerion angustifolium* L. Holub was performed to evaluate harvest-stage effects. Phytochemical characterisation was performed using spectrophotometric assays and high-performance liquid chromatography (HPLC) analyses. Among the tested extraction methods, 75% (*v*/*v*) methanol in water was the most effective conventional solvent, and ultrasound-assisted extraction yielded the highest overall TPC (total phenolic content), TFC (total flavonoid content), and RSA (radical scavenging activity) values. Vegetation stage analysis of *C. angustifolium* L. Holub revealed significant variation in phenolic content and antioxidant activity, with the highest levels observed at the beginning of the flowering. Antiproliferative activity was assessed against five cancer cell lines (4T1, A549, Caki-1, HCT116 and MCF7), while HEK-293 cells were used as an immortalised non-cancerous reference model for general cytotoxicity evaluation. Linear mixed-model analysis confirmed a significant incubation-time effect in all tested cancer cell lines, with IC_50_ values generally decreasing after prolonged exposure. Statistically significant F2-F3 differences were plant-dependent. The 30% (*v*/*v*) methanol in water fraction (F2), enriched in oenothein B in *C. angustifolium* L., showed stronger antiproliferative activity, whereas the 60% (*v*/*v*) methanol in water fraction (F3) showed stronger activity in *Quercus robur* L., *Juglans nigra* L., *Juglans regia* L., and *Solidago canadensis* L. These findings indicate that antiproliferative activity was associated with the qualitative and quantitative composition of the selected phenolic-rich SPE fractions rather than with a single universal fraction effect. All tested fractions exhibited lower cytotoxicity toward HEK-293 cells under the applied conditions; however, claims of selectivity should be confirmed using additional normal or primary cell models. Overall, the findings clarify the role of extraction strategy, harvest stage and targeted fractionation in linking phenolic composition with biological activity.

## 1. Introduction

Medicinal plants are a rich source of biologically active secondary metabolites with diverse pharmacological properties, including antioxidant and anticancer activities. Among these compounds, phenolic acids, flavonoids, and ellagitannins have received particular attention due to their ability to modulate oxidative stress, regulate cellular signalling pathways, and inhibit cancer cell proliferation [1,2]. Plant extracts also contain other bioactive compound classes, including terpenoids, fatty acids, phytosterols and carotenoids, which may contribute to biological effects [3]. Therefore, the present study uses the narrower term “phenolic profiling” when referring to the targeted analytical focus of this work. The biological activity of plant extracts is closely related to both the qualitative and quantitative composition of the phytochemicals they contain.

The extraction of phenolic compounds from plant material depends heavily on the chosen extraction strategy. Factors such as solvent polarity, extraction time, temperature, and sample matrix properties significantly impact the extraction efficiency and stability of compounds. Aqueous alcohol solvents are widely used due to their ability to extract a wide range of phenolic compounds with different polarities, while purely aqueous or non-polar solvents often result in lower extraction yields [4]. In recent years, advanced extraction techniques, such as ultrasound-assisted extraction, have been increasingly applied to improve mass transfer and enhance the extraction of phenolic compounds and their associated antioxidant activity [5]. Other methods, including hydrolysis-assisted and fermentation-assisted extractions, can further modify the phytochemical composition by releasing bound polyphenolic compounds or by inducing biochemical changes in the plant matrix [6,7]. The separation, identification, and characterisation of plant extracts require additional analytical methods. Spectrophotometric assays are commonly used for the determination of total phenolic and flavonoid content, as well as radical scavenging activity, while chromatographic methods, such as high-performance liquid chromatography, allow the identification and quantification of individual compounds [2]. These analytical methods are essential for establishing the relationship between phytochemical composition and biological activity.

Despite extensive studies on plant extracts, identifying specific groups of compounds responsible for anticancer activity remains a major challenge, especially given the complexity of plant matrices and the potential synergistic interactions between compounds [8,9]. Most studies have focused on crude extracts, limiting the ability to distinguish the contributions of different phytochemical classes. Solid-phase extraction is a powerful tool to address this limitation by separating crude extracts into physicochemically distinct fractions based on polarity, thereby enabling a more accurate assessment of biological activity [10]. In our previous study [11], 75% (*v*/*v*) methanol in water extracts of selected medicinal plants rich in ellagic acid derivatives showed significant antiproliferative activity against multiple cancer cell lines. However, that study evaluated crude extracts and did not determine whether the observed activity was associated with phenolic acid- or ellagitannin-rich fractions, flavanoid-rich fractions, or the unfractionated extracts as a whole. The five species included in the present study were selected because they are medicinally relevant plant materials with phenolic-rich profiles and because their crude extracts previously showed reproducible in vitro antiproliferative activity [11].

The selected species also have documented medicinal or traditional relevance. *Chamaenerion angustifolium* L. has been traditionally used for inflammatory, gastrointestinal, urinary and skin-related conditions [12,13]. *Quercus robur* L. is a tannin- and phenolic-rich oak species with documented medicinal relevance within the genus *Quercus*, including traditional astringent applications, and *Q. robur* L. leaves have been reported to contain tannins and flavonoids with antioxidant, antimicrobial and wound-healing-related potential [14,15]. *Juglans regia* L. and *Juglans nigra* L. are phenolic-rich walnut plant materials with reported antioxidant, antimicrobial and anti-inflammatory potential [16,17], and *Solidago canadensis* L. has been used in phytotherapeutic contexts related to urinary and inflammatory conditions [18]. Thus, the selected species were not chosen solely because of their previously observed antiproliferative activity, but also because they represent botanically diverse, medicinally relevant, phenolic-rich plant materials.

Although these species are taxonomically unrelated and differ in harvested plant part, this diversity was intentionally retained to test whether fraction-dependent activity patterns are plant-specific rather than universal. In addition to extraction and fractionation, other factors such as species and vegetation stage also significantly influence the phytochemical composition of medicinal plants. It has been shown that the vegetation stage has a significant impact on the accumulation of phenolic compounds, as the biosynthesis of secondary metabolites is closely related to plant growth, environmental conditions and phytochemical processes [19]. Such variability may affect not only antioxidant capacity but also biological activity; therefore, it is important to consider plant development when assessing the potential anticancer activity. Furthermore, linking phytochemical composition to biological activity requires not only qualitative observations but also appropriate statistical evaluation. Differences between fractions or processing conditions are often subtle, and apparent trends may not reflect statistically significant effects. Therefore, the integration of statistical methods, including analysis of variance, mixed-model analysis, and correlation analysis, is essential for reliable interpretation of relationships between phytochemical composition and biological activity in complex plant extracts.

The main objective of this study was to determine whether phenolic-rich C18 solid-phase extraction (SPE) fractions with different polarity and composition are associated with different antiproliferative effects in selected medicinal plant extracts. The central hypothesis was that reducing the complexity of previously active crude extracts into phenolic-rich fractions would reveal plant-dependent differences in biological activity that are not evident from crude extract screening alone. To support this objective, the study was organized into three complementary but distinct parts: (1) C18 SPE fractionation of the previously active 75% (*v*/*v*) methanol in water extracts followed by phenolic characterization and in vitro antiproliferative evaluation; (2) comparison of extraction strategies in a subset of representative species to show how extraction method affects phenolic recovery and antioxidant capacity, rather than to re-optimise the fractionation solvent; and (3) vegetation-stage profiling of *C. angustifolium* L. to evaluate how harvest stage influences phenolic compound accumulation. This design clarifies that extraction-method comparison, vegetation-stage profiling and SPE fractionation answer related but separate questions within the broader aim of linking phenolic composition with biological activity.

## 2. Materials and Methods

### 2.1. Chemicals and Reagents

Gallic acid (95%), DMEM medium, RPMI medium, trypsin-EDTA (0.25%), fetal bovine serum (FBS), penicillin-streptomycin (10,000 U/mL), and L-glutamine (200 mM) were obtained from Thermo Scientific (Waltham, MA, USA). Ellagic acid (95%), vanillic acid (97%), chlorogenic acid (95%), ferulic acid (99%), 2-hydroxycinnamic acid (97%), trans-sinapic acid (99%), trans-p-coumaric acid (99.7%), 3,4-dihydroxybenzoic acid (97%), dichloromethane (99% (vol.)), ethyl acetate (99% (vol.)), sodium hydroxide (95%), glycerol (99.5% (vol.)) and DPPH (2,2-diphenyl-1-picrylhydrazyl) (99%) were purchased from Sigma Aldrich (St. Louis, MO, USA). Rutin (95%), acetonitrile (99%), trifluoracetic acid (99% (vol.)), Folin–Ciocalteu’s phenol reagent (2N) and MRS broth with Tween 80 (99%) were obtained from Merck (Darmstadt, Germany). Sodium carbonate (p.a.), acetic acid (80%; (*v*/*v*), hydrochloric acid (80% (vol.) and methanol (p.a.) were obtained from Chempur (Piekary Śląskie, Poland). Dimethyl sulfoxide (99% (*v*/*v*)) and hexamethylenetetramine (99%) were obtained from Carl Roth (Karlsruhe, Germany), while aluminum chloride (99.0%) and sodium acetate (99%) were obtained from Alfa Aesar (Karlsruhe, Germany). Hexane (99% (vol.)) was obtained from Thermo Scientific (Waltham, MA, USA), and CellTiter 96^®^ AQ_ueous_ one solution reagent was obtained from Promega (Madison, WI, USA). Bidistilled water prepared in the laboratory using a Fistreem Cyclon bidistillator (Fistreem Cyclon, Cambridge, UK).

### 2.2. Plant Material

For this study, five medicinal plants (*Chamaenerion angustifolium* L. Holub, *Quercus robur* L., *Juglans nigra*, L., *Solidago canadensis* L., and *Juglans regia* L.) were collected at various vegetation stages in the Kaunas Botanical Garden of Vytautas Magnus University from May to September 2023, using the plant parts and phenological stages listed in Table 1. All samples were obtained from the same botanical garden collection site, and collection dates differ because sampling was based on the relevant plant part and phenological stage for each species. The identity of all collected plant samples was confirmed by Dr. Ona Ragažinskienė, a botanist at the Kaunas Botanical Garden Herbarium (international identification code KAUN), for future verification. Based on our previous screening study [11], these five species were selected because their crude (75% (*v*/*v*) methanol in water) extracts showed pronounced and reproducible antiproliferative effects in vitro against several cancer cell lines and because they represent medicinal plant materials rich in phenolic compounds. The selected plant parts were aerial parts (*Herba*) of *C. angustifolium* L. and *S. canadensis* L., *Q. robur* L. leaves, and *J. nigra* L. and *J. regia* L. pericarps. Different fresh plant parts were collected and dried in the dark at room temperature for 7–10 days. A total of nine plant samples were used in this study.

Table 1 lists all medicinal plants and their parts used in the study. Only *C. angustifolium* L. was intentionally sampled across five vegetation stages for the harvested-stage comparison, and the remaining species were collected at the plant stage and plant part relevant to the previously observed crude extract activity [11].

The moisture content of each medicinal plant sample was determined using a PBM-53 Moisture Balance (Adam Equipment, Kingston, Milton Keynes, United Kingdom) according to the manufacturer’s recommendations.

### 2.3. Experimental Design and Extraction Procedures

**Figure 1:**
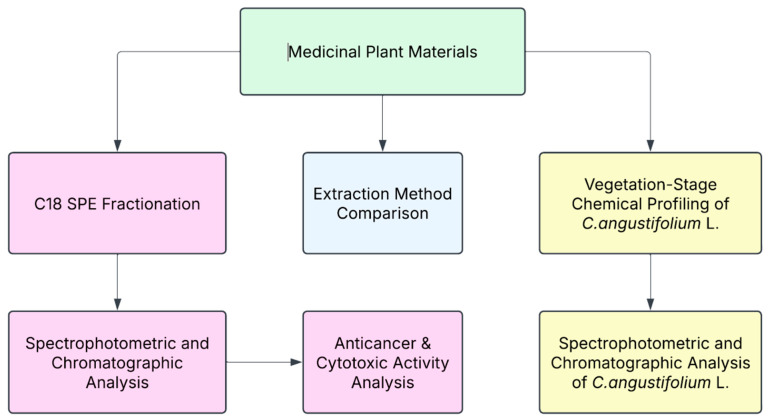
Experimental workflow overview and plant sample allocation. The diagram summarises three parallel experimental workflows: (1) C18 solid-phase extraction (SPE) fractionation for chemical profiling and in vitro anticancer activity evaluation; (2) extraction-method comparison of selected plant samples; and (3) vegetation stage-dependent chemical profiling performed only for *C. angustifolium* L. Arrows indicate sample allocation, not linear experimental sequence.

This section describes the general experimental design, sample allocation, and extraction strategies applied in the study. Detailed descriptions of spectrophotometric, chromatographic, and biological assays are provided in Section 2.4, Section 2.5, Section 2.6 and Section 2.7. Figure 1 summarises the three complementary workflows and indicates sample allocation; the arrows indicate allocation to analytical workflows rather than a single linear experimental sequence.

#### 2.3.1. Study Design and Sample Allocation

Plant samples were divided into three complementary workflows based on the study objectives. First, to investigate which phenolic-rich SPE fraction(s) of the crude 75% (*v*/*v*) methanol in water extracts contributed most to the antiproliferative activity previously observed in our screening study [11], five plant samples (*C. angustifolium* L. (beginning of the flowering vegetation stage), *Q. robur* L., *J. nigra* L., *S. canadensis* L. and *J. regia* L.) were selected for fractionation. The crude extracts prepared from these samples were fractionated by C18 SPE to obtain physicochemically distinct fractions, which were subsequently characterised and tested for antiproliferative activity.

Second, to compare how the extraction strategy affects phenolic recovery and antioxidant capacity, an additional subset of three representative plant samples (*C. angustifolium* L. (beginning of the flowering vegetation stage), *Q. robur* L. and *J. nigra* L.) was selected. These species were chosen because they represented different plant material types and showed high phenolic content and reproducible activity in the previous screening study [11]. This subset was used to compare method-dependent phenolic recovery in representative matrices and was not intended as a full five-species extraction optimisation study. These samples were processed using several extraction methods, including solvent extraction with increasingly polar solvents, ultrasound-assisted extraction, hydrolysis-assisted extraction, and fermentation-assisted extraction. This workflow was intended to evaluate method-dependent phenolic profiles and was not used to validate or replace the 75% (*v*/*v*) methanol in water extraction solvent used for SPE fractionation, which had already been selected based on the previously crude-extracts study.

Third, a vegetation-stage experiment was performed only for *C. angustifolium* L. samples. Aerial parts (*Herba*) were collected at five vegetation stages (intensive growth, flower bud development, beginning of flowering, massive flowering and end of flowering; see Table 1) to assess stage-dependent changes in phenolic compound accumulation. All stage-specific samples were processed using the same extraction and analytical methods to enable direct comparison of spectrophotometric and chromatographic profiles at different vegetation stages.

#### 2.3.2. Preparation of Plant Material

After drying, the plant material was ground in an electric grinder to a particle size of 1–3 mm and stored in airtight containers protected from light until extraction. The amount of material required for each extraction procedure was accurately weighed to ensure comparability between samples and extraction methods.

#### 2.3.3. SPE Fractionation for Antiproliferative Activity Testing

Crude extracts (75% (*v*/*v*) methanol in water) were prepared as described previously [11] and subsequently fractionated by solid-phase extraction to obtain chemically distinct fractions for further analyses and anticancer activity studies. Fractionation was performed using CHROMABOND^®^ C18 ec cartridges (octadecyl-modified silica, end-capped; 6 mL/500 mg; Macherey-Nagel, Düren, Germany). The overall SPE workflow (conditioning, sample loading, and stepwise elution) followed a previously published C18 SPE scheme, with modifications to match the present samples and fractionation plan [2]. Before loading samples, the cartridges were conditioned with 20 mL of 100% methanol, followed by 20 mL of bidistilled water. The extract (5 mL) was diluted with bidistilled water (0.05% (*v*/*v*) trifluoracetic acid) to 50 mL and loaded into the cartridge under vacuum. After loading the sample, the cartridge was drained and dried under vacuum.

Fractions were collected by stepwise elution with increasing methanol concentration: 5 mL of bidistilled water (0%; F1), followed by 5 mL of 30% (*v*/*v*) methanol in water (F2), 5 mL of 60% (*v*/*v*) methanol in water (F3), and 5 mL of 100% methanol (F4). F1 was treated as the aqueous wash fraction containing unretained or weakly retained highly polar compounds, while F4 represented the strongest organic eluate. All four eluates were initially screened by spectrophotometric and chromatographic analyses. Under the applied analytical conditions, no relevant phenolic, chromatographic or antioxidant signals were detected in F1 and F4, whereas F2 and F3 contained detectable phenolic compounds and antioxidant activity. Therefore, only F2 and F3 were selected for cell viability testing, as these fractions represent the analytically confirmed phenolic-rich fractions targeted by the study and could be transferred into a cell-compatible solvent system after methanol removal. The exclusion of F1 and F4 from the antiproliferative assay was therefore based on the absence of detectable relevant phenolic/chromatographic signals under the applied methods and the predefined focus on F2/F3 phenolic-rich fractions. However, this does not exclude the possible presence of compounds below the detection limits or compounds not detectable by the applied analytical methods. For selected fractions, 4 mL of each eluate was evaporated to remove methanol using a rotary evaporator (Heidolph™ VV Micro, Heidolph Instruments, Schwabach, Germany) with a heated glycerol bath (80 °C). The exposure time at this temperature was short (approximately 8 min) and was used only for solvent removal; nevertheless, possible heat-related transformation of sensitive constituents is acknowledged as a limitation. After evaporation, the residue was dissolved in 1 mL 5% (*v*/*v*) dimethyl sulfoxide (DMSO) in water, as higher methanol concentrations are not compatible with cell-based assays [20]. A matched 5% (*v*/*v*) DMSO in water solvent control was included in the cell viability assay to verify that the solvent system did not negatively affect cell growth under the applied conditions. Fractions were stored at 4 °C in a refrigerator for further analysis.

#### 2.3.4. Extraction Using Solvents of Increasing Polarity

To compare extraction efficiency across solvents differing in polarity and composition, a series of extraction solvents was applied, including non-polar (100% hexane), moderately polar (100% dichloromethane and 100% ethyl acetate), and polar solvents (100% methanol, and 100% bidistilled water), as well as aqueous alcohol mixtures (75% (*v*/*v*) ethanol in water, 75% (*v*/*v*) methanol in water), which are commonly used for phenolic compound extraction [21,22]. Non-polar and moderately polar solvents were included to provide a broader polarity comparison and to verify whether non-phenolic or weakly polar constituents were detectable chromatographically; they were not intended as primary solvents for phenolic extraction. Extractions were performed in parallel, using fresh plant material for each solvent. 0.5 g of plant material was extracted with 20 mL of the respective solvent by shaking in an orbital shaker (Lin-Pro, Labbox labware, Barcelona, Spain) for 24 h at 200 rpm at room temperature. The 24 h extraction period was chosen to maintain comparability with the previous crude-extract workflow and to ensure exhaustive maceration; however, potential oxidation or degradation during prolonged extraction is acknowledged as a limitation. The resulting extracts were filtered through filter paper, followed by a 0.22 µm membrane filter, and stored at 4 °C until further analysis. All extractions were performed under the same controlled conditions (room temperature, solvent-to-solid ratio, and extraction time) to ensure reproducibility and comparability between samples. Due to limitations in solvent miscibility in aqueous assays, spectrophotometric measurements were performed on methanol, ethanol, and water-based extracts. High-performance liquid chromatography (HPLC) was used to analyse all solvent extracts and compare chromatographic profiles across the polarity series.

#### 2.3.5. Ultrasound-Assisted Extraction

Ultrasound-assisted extraction (UAE) was performed using 75% (*v*/*v*) methanol in water as the extraction solvent, as previously reported [23], with modifications. In this method, 0.5 g of plant material was mixed with 20 mL of 75% (*v*/*v*) methanol in water and sonicated in an ultrasonic batch at room temperature (~25 °C) for 1 h. After extraction, the mixture was filtered through a 0.22 µm membrane filter and stored at 4 °C for further analysis.

#### 2.3.6. Hydrolysis-Assisted Extraction

Alkaline hydrolysis-assisted extraction was performed according to [24], with modifications, while acidic hydrolysis-assisted extraction was performed according to [25], with modifications. The alkaline and acidic protocols were derived from different procedures in the literature and therefore differed in temperature: alkaline hydrolysis was performed at room temperature, whereas acidic hydrolysis was performed at 60 °C. Consequently, the results should be interpreted as a comparison of complete hydrolysis-assisted extraction conditions rather than as an isolated comparison of alkaline and acidic pH effects only, because temperature may have contributed to the observed differences. After hydrolysis-assisted extraction, a SPE clean-up procedure was adapted from [26,27], with modifications. For alkaline hydrolysis-assisted extraction, 0.5 g of plant material was mixed with 20 mL of 75% (*v*/*v*) methanol in water containing 0.5 M NaOH. The mixture was incubated for 1 h at room temperature in the dark, continuously shaking at 200 rpm. For acidic hydrolysis-assisted extraction, a 0.5 g of plant material was mixed with 20 mL of 75% (*v*/*v*) methanol containing 0.5 M HCl. The mixture was incubated for 1 h at 60 °C in the dark. After extraction, each mixture was cooled to room temperature and centrifuged at 5000 rpm for 10 min at room temperature. The supernatant was collected. The alkaline hydrolysate was adjusted to pH 3.0 using 6 M hydrochloric acid, while the acid hydrolysate was adjusted to pH 3.0 using 5 M sodium hydroxide. Before the SPE loading, 2 mL of clarified supernatant was diluted to 10 mL with acidified water (pH 3.0; prepared by adjusting distilled water with 0.1 M HCl), to reduce the methanol concentration. C18 SPE cartridges (CHROMABOND^®^; octadecyl-modified silica, end-capped; 6 mL/500 mg; Macherey-Nagel, Germany) were conditioned with 5 mL 100% (*v*/*v*) methanol followed by 5 mL of acidified water (pH 3.0). The diluted extract was loaded onto the cartridge under vacuum. The cartridge was washed with 5 mL of acidified water (pH 3.0), and phenolic compounds were eluted with 5 mL of 75% (*v*/*v*) methanol in water. The eluates were collected, filtered through 0.22 µm membrane filter and stored at 4 °C for further analysis.

#### 2.3.7. Fermentation-Assisted Extraction

Fermentation-assisted extraction was performed according to the solid-phase lactic acid fermentation method described by Damulienė et al. [28], with modifications. The 48 h fermentation period was selected based on a previous solid-phase fermentation approach and was used as a preliminary condition for comparing fermented and non-fermented plant material. Briefly, 5 g of plant material was moistened with 5 mL of sterile bidistilled water and left to equilibrate for 2 h. Then, a sterile glucose solution (1.5 g glucose dissolved in 2.5 mL water) was added. Fermentation was initiated with *Lacticaseibacillus rahamnosus* GG (ATCC 53103) (Gefilus, Valio Ltd., Helsinki, Finland). The culture was revived in MRS broth supplemented with Tween 80 and incubated at 37 °C until OD_600_ reached 0.730, after which 800 µL of bacterial suspension (≈2.9 × 10^9^ CFU/mL) was added to the hydrated plant material under sterile conditions. The mixture was incubated at 37 °C for 48 h. After fermentation, metabolites were extracted using 75% (*v*/*v*) methanol in water at a 1:10 (*w*/*v*) solid-to-solvent ratio (100 mL solvent per 10 g wet material equivalent), with shaking at 200 rpm for 3 h at room temperature. The resulting extracts were filtered through a 0.22 µm membrane filter. Samples were stored at 4 °C in a refrigerator for further analysis. Because pH dynamics and sterility-control samples were not systematically evaluated in this exploratory fermentation workflow, the resulting fermentation-related changes are interpreted with caution and acknowledged as a limitation.

### 2.4. Spectrophotometric Analysis

Total phenolic content, total flavonoid content and free radical scavenging activity were quantified by UV-Vis spectrophotometry (Milton Roy Spectronic, Ivyland, PA, USA). TPC was determined by the Folin–Ciocalteu method, and TFC by the colourimetric AlCl_3_ method. Both methods were applied with minor modifications as described by Bimbiraitė-Survilienė et al. [21]. Antioxidant capacity was assessed using the DPPH radical scavenging assay [29]. For all measurements, reagent blanks prepared under identical reaction conditions were used for background correction, and each sample was analysed in triplicate.

For TPC analysis, 100 µL of extract was mixed with 100 µL of 2 N Folin–Ciocalteu reagent and 3000 µL of 4% (*v*/*v*) sodium carbonate solution. After 30 min incubation at room temperature, absorbance was measured at 760 nm. For TFC determination, 80 µL of extract was mixed with AlCl_3_ reagent solution prepared in methanol containing acetic acid, hexamethylenetetramine, and aluminium chloride. The mixture was incubated for 30 min at 4 °C to maintain reaction stability and minimise non-specific oxidation during colour development, and absorbance was measured at 407 nm. For the RSA assay, 77 µL of extract was added to 3000 µL of DPPH solution (0.1 mM), and the mixture was incubated in the dark at room temperature for 15 min before absorbance was measured at 515 nm.

Quantification was performed using rutin as a common reference standard. A rutin stock solution (1 mg/mL) was prepared in 75% (*v*/*v*) methanol in water, and working standards were obtained by serial dilution to appropriate concentration ranges for each assay. Calibration curves were constructed from mean absorbance (*n* = 3) and showed linear relationships over the entire assay range. The resulting regression equations were fitted to calculate TPC, TFC and RSA, expressed as mg rutin equivalents (RE) per gram of dry raw material. Rutin was retained as a common internal reference to allow direct comparison across the different assays and fractions in this study. However, because gallic acid is a more conventional standard for TPC assays, while Trolox or ascorbic acid are commonly used as reference standards for DPPH radical-scavenging assay, the use of rutin equivalents limits direct comparison with some literature values and is acknowledged as a methodological limitation.

Limits of detection (LOD) and quantification (LOQ) were determined based on the standard deviation of replicate measurements (*n* = 3) and the slope of calibration curves, using the equations LOD = 3.3 σ/S and LOQ = 10 σ/S, where σ represents the standard deviation of the response, and S is the slope of the calibration curve. The calculated LOD/LOQ values were 0.009/0.027 mg/mL for TPC, 0.006/0.017 mg/mL for TFC, and 0.006/0.017 mg/mL for RSA.

### 2.5. HPLC UV-Vis and HPLC-ED System Analysis

Phenolic compounds in the plant extracts and fractions were separated and identified using two chromatographic systems: a high-performance liquid chromatography (HPLC) with UV-Vis (ultraviolet–visible) detection (HPLC UV-Vis) and an HPLC system coupled with electrochemical detection (ED). The two systems were used for complementary purposes rather than as interchangeable quantification methods. HPLC UV-Vis was used to visualise overall chromatographic profiles and compare retention times with externally injected standards, whereas HPLC-ED provided sensitive detection of redox-active phenolic compounds. Both analyses were performed using chromatographic conditions adapted from our previous works [11,22,29], as described below.

UV-Vis spectroscopy-based separation was carried out on modular HPLC setup consisting of binary pump (Agilent 1100 HPLC G1312A, Santa Clara, CA, USA), a manual injector (Rheodyne™ 7725, Santa Clara, CA, USA), and a reversed-phase RP-18 column (125 mm × 4 mm, LiChrospher^®^ 100 RP-18, 5 µm; Merck, Darmstadt, Germany) coupled to a UV detector (Linear UVIS 200, Waltham, MA, USA). Samples were injected using a 25 µL Hamilton^®^ syringe (model 1702 RNR; Hamilton Company, Reno, NV, USA) with a 20 µL injection volume. The mobile phase consisted of bidistilled water containing 0.05% (*v*/*v*) trifluoracetic acid (Solvent A) and methanol containing 0.05% (*v*/*v*) trifluoracetic acid (Solvent B). Elution was performed at a flow rate of 0.75 mL/min using the following gradient: 0–10 min, 0–25% B; 10–38 min, 25–60% B; 38–40 min, 60–95% B; followed by an isocratic hold at 95% B for 5 min. Detection was conducted at 265 nm. Data acquisition and processing were performed using DataApex Clarity Lite software (version 2.6.6.574). Compounds were identified by comparing retention times of externally injected standards.

Antioxidant electrochemical profiling was performed using a gradient HPLC system (ESA, Chelmsford, MA, USA) equipped with two ESA 582 pumps operating in high-performance gradient mode and a CoulArray electrochemical detector (model 5600). Separation was performed on a reversed-phase C18 column (125 mm × 4 mm, LiChrospher^®^ 100 RP-18e, 5 µm; Merck, Darmstadt, Germany). Samples (10 µL) were manually injected using a 25 µL Hamilton^®^ syringe (model 1702 RNR), and the flow rate was maintained at 0.4 mL/min throughout the analysis. The detector cell array was operated at 300, 500, and 700 mV. The dominant response (the highest peak recorded in a single cell) was used for quantification. Mobile phase A consisted of 50 mM sodium dihydrogen phosphate buffer (pH 3) containing 1% (*v*/*v*) methanol, whereas mobile phase B comprised 100 mM sodium dihydrogen phosphate buffer (pH 3), acetonitrile, and methanol (30:60:10, *v*/*v*/*v*). The gradient program started at 25% B (5 min), increased to 60% by 38 min, reached 95% by 45 min, and then returned to 5% at 58 min, remaining at 5% until the column was fully equilibrated after 60 min. Data were processed using CoulArray^®^ software (version 1.02).

In both systems, external standards were injected under identical chromatographic conditions to determine retention times and support compound identification. In HPLC UV-Vis analysis, compounds were identified primarily by matching retention times with externally injected standards at the selected detection wavelength. UV spectra were not used as an additional criterion for identification because detection was performed at a fixed wavelength. In HPLC-ED analysis, retention-time matching was used together with the electrochemical response of redox-active compounds as supportive evidence. The standards included 3,4-dihydrobenzoic acid, trans-p-coumaric acid, chlorogenic acid, gallic acid, caffeic acid, syringic acid, rutin, vanillic acid, trans-sinapic acid, ferulic acid, ellagic acid, and 2-dihydrocynammic acid. Because individual calibration curves were not constructed for each identified compound, concentrations were calculated relative to a rutin calibration curve and expressed as mg rutin equivalents per gram of dry raw material. Therefore, the reported HPLC values should be interpreted as semi-quantitative estimates rather than absolute compound concentration. LOD and LOQ for both HPLC methods were determined in the same calculations as described above in Section 2.4. For HPLC UV-Vis analysis, the LOD and LOQ values were 0.001/0.003 mg/mL and for HPLC-ED analysis, the LOD and LOQ values were 0.003/0.009 mg/mL.

### 2.6. Cell Culture and Effect on Cell Viability

#### 2.6.1. Cell Lines and Culture Conditions

The antiproliferative effects of medicinal plant extract fractions were evaluated using five cancer cell lines: 4T1 (mouse mammary carcinoma), A549 (human lung adenocarcinoma), Caki-1 (human kidney carcinoma), HCT116 (human colon carcinoma), and MCF7 (human breast adenocarcinoma). In addition, HEK-293 (human embryonic kidney) cells were included as an immortalised non-cancerous reference cell line for general cytotoxicity evaluation. HEK-293 cells do not represent normal tissue or a tissue-matched primary control for the cancer cell lines tested; therefore, they were used only to provide a preliminary reference for non-cancerous cell sensitivity under the in vitro conditions used. All cell lines were immortalised lines obtained from the American Type Culture Collection (ATCC, Manassas, VA, USA). Cells were thawed from frozen stocks and maintained as adherent monolayers under standard culture conditions prior to experiments. Depending on the cell type, cells were cultured in RPMI or DMEM medium supplemented with 10% FBS and 1% penicillin-streptomycin; DMEM-based cultures were additionally supplemented with 1% L-glutamine. Cells were passaged every 2–3 days, and experiments were initiated after stable growth was reestablished following thawing (typically after 2–3 weeks). All cell culture conditions and the MTS-based viability assessment were performed exactly as described in our previous study [11].

#### 2.6.2. MTS Cell Proliferation Assay

Cell viability was assessed using the MTS colorimetric assay, which quantifies metabolic activity by reducing the tetrazolium compound MTS (3-(4,5-dimethylthiazol-2-yl)-5-(3-carboxymethoxyphenyl)-2-(4-sulfophenyl)-2H-tetrazolium) to a soluble formazan product by viable cells [30,31]. Cells were plated in 96-well plates at a density adjusted to maintain similar confluence during the measurement: 8000 cells/well for 24 h exposure, 4000 cells/well for 48 h exposure, and 2000 cells/well for 72 h exposure. Plant extract fractions (1–18 µL per well) were added together with culture medium (RPMI or DMEM, 182–199 µL) to a final volume of 200 µL per well. Untreated cells cultured in medium alone served as the positive growth control (200 µL), and wells containing only medium served as the background control. Each concentration was tested in triplicate wells, and the experiment was repeated independently to confirm reproducibility. Test concentrations were prepared by serial dilution of fraction stock solutions in culture medium, maintaining the same solvent concentration in the treatment and solvent control wells. A 5% (*v*/*v*) DMSO in water control was included to verify that the solvent did not negatively affect cell growth under the applied conditions.

The 96-well plates were incubated at 37 °C in a humidified environment containing 6.0% CO_2_ for 24, 48 and 72 h (CelCulture^®^ CO_2_ incubator, Esco Lifesciences, Singapore). After the incubation period, the medium containing extract fractions was removed and replaced with 100 µL of fresh RMPI or DMEM. Then, 20 µL of CellTiter 96^®^ AQ_ueous_ one solution reagent was added to each well. Then, 96-well plates were incubated for 3 h at 37 °C (6.0% CO_2_), and absorbance was measured at 492 nm using Biosan HiPo MPP-96 microplate photometer (Biosan, Riga, Latvia). Dose–response curves were generated by plotting the concentration of the extract fraction, expressed as mg dry raw material (DRM) equivalents per mL (mg DRM/mL), against cell viability (%). IC_50_ values were calculated from the corresponding regression models and reported as the concentration of dry raw material equivalent required to inhibit 50% of cell viability. Because this unit reflects the amount of original dried plant material present in the tested fraction rather than purified compound concentration, the IC_50_ values are most appropriate for within-study comparison and are not directly comparable with IC_50_ values reported for isolated compounds or standardised extracts.

### 2.7. Statistical Analysis

All statistical analyses were performed using IBM SPSS Statistics, version 31.0.2.0 (SPSS Inc., Chicago, IL, USA). All assays were performed in triplicate (*n* = 3), and results were expressed as mean ± relative standard deviation (RSD). IC_50_ values were expressed as mg dry raw material equivalent per mL (mg DRM/mL) and log_10_-transformed prior to statistical analysis. Values reported as not determined (ND), where 50% inhibition was not reached within the tested concentration range, were excluded from the continuous IC_50_ analysis but included in a categorical analysis indicating whether 50% inhibition was reached.

The effects of plant species, SPE fraction, incubation time, and cancer cell line on log_10_-transformed IC_50_ values were evaluated using linear mixed-effects models. Plant species, fraction, incubation time, cancer cell line, and the plant species × fraction interaction were included as fixed effects, while sample identity was included as a random intercept. Significant effects were followed by Holm-adjusted post hoc comparisons. Plant-specific F2-F3 comparisons were performed using matched cell line-incubation time pairs and analysed using paired-samples *t*-tests or Wilcoxon signed-rank tests, depending on the distribution. The frequency of calculable IC_50_ values between F2 and F3 was compared using McNemar’s exact test. Plant-specific comparisons were adjusted for multiple testing using the Holm method.

Differences among vegetation stages of *C. angustifolium* L. were evaluated using one-way analysis of variance (ANOVA), followed by Tukey’s honestly significant differences (HSD) post hoc test. Where Tukey HSD post hoc testing was applied, statistically significant group differences are indicated in the corresponding table using superscript letters. Differences in chemical composition parameters between fractions were evaluated using independent sample *t*-tests, while changes between conventional 75% (*v*/*v*) methanol in water extraction and fermentation-assisted extraction were evaluated using Welch’s *t*-test.

Associations between chemical composition parameters and antiproliferative activity were assessed using Spearman’s rank correlation coefficients between TPC, TFC, RSA, chromatographic peak areas, and log_10_-transformed IC_50_ values. For chromatographic data, total peak area was used in the statistical analysis because it represents the summed signal of all detected peaks, including peaks that were not individually identified or quantified by external standards. Only IC_50_ values that could be calculated were included. *p*-values from chemical parameters-IC_50_ correlation analyses were adjusted using the Benjamini–Hochberg false discovery rate procedure. Correlation analyses were considered exploratory, and a *p*-value < 0.05 was considered statistically significant.

## 3. Results

The results are presented according to the three complementary workflows described in Section 2.3 and Figure 1: vegetation-stage profiling of *C. angustifolium* L., extraction-method comparison in selected representative species, and C18 SPE fractionation followed by phenolic characterisation and antiproliferative evaluation. These workflows were not intended as a single factorial experiment combining all plant species, plant parts, phenological stages, extraction methods and biological assays. Instead, each workflow addresses a separate question within the broader aim of linking phenolic composition with biological activity.

### 3.1. Vegetation Stage-Dependent Variation in C. angustifolium L.

#### 3.1.1. Changes in TPC, TFC and RSA Across Vegetation Stages

The differences in TPC, TFC and RSA in *Chamaenerion angustifolium* L. Holub across different vegetation stages are summarised in Table 2. The moisture content of the room-dried samples ranged from 5.12% to 6.87%, confirming that the samples were properly dried and stored before extraction. Water content was taken into account to present the results on a dry-matter basis.

As shown in Table 2, the accumulation of bioactive compounds in *C. angustifolium* L. varied significantly depending on the stage of vegetation. One-way ANOVA revealed statistically significant differences among stages for TPC (F(4, 10) = 2547.48, *p* < 0.001), TFC (F(4, 10) = 3228.53, *p* < 0.001), and RSA (F(4, 10) = 8892.74, *p* < 0.001).

The highest TPC (116.8 ± 2.41 mg RE/g), TFC (20.29 ± 0.94 mg RE/g), and RSA (101.49 ± 1.98 mg RE/g) values were observed at the beginning of flowering, differing significantly from all other vegetation stages (Tukey HSD, *p* < 0.001). Moderate levels were determined during intensive growth and bud development, while a pronounced decrease in all measured parameters was determined during the massive flowering vegetation stage, which showed significantly lower values than in all other stages (*p* < 0.001). At the end of flowering, TPC, TFC, and RSA values increased slightly compared to massive flowering but remained significantly lower than those observed during the early reproductive stages (*p* < 0.001). These results indicate a clear vegetation stage-dependent pattern in the antioxidant potential of *C. angustifolium* L. extracts.

#### 3.1.2. Stage-Specific Differences in Chromatographic Profiles

**Figure 2:**
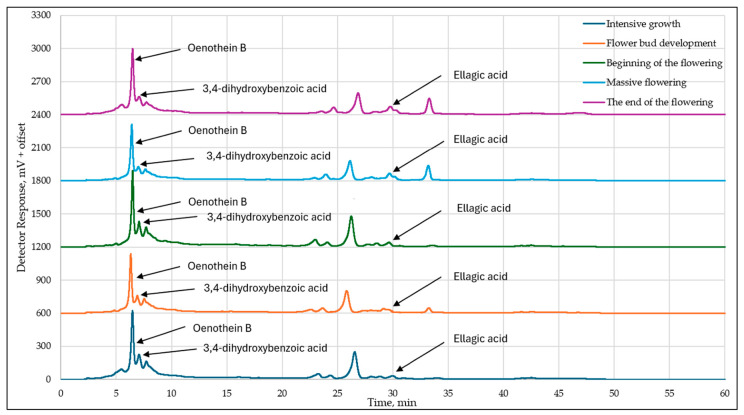
Stage-specific HPLC UV-Vis chromatographic profiles of *C. angustifolium* L. extracts obtained at different vegetation stages. Major identified phenolic compounds are indicated, while signal intensities are presented with vertical offset for clarity.

In order to further explain the vegetation stage-dependent changes in the phytochemical composition of *C. angustifolium* L., extracts were analysed using HPLC with UV-Vis and electrochemical detection. HPLC UV-Vis chromatograms corresponding to the vegetation stages of intensive growth, flower bud development, beginning of the flowering, massive flowering and end of the flowering stages are shown in Figure 2.

Clear qualitative and semi-quantitative differences in chromatographic profiles were observed across different vegetation stages. The beginning of the flowering was characterised by the most distinct peaks of oenothein B, 3,4-dihydroxybenzoic acid and ellagic acid, corresponding with the highest values of TPC, TFC, and RSA determined spectrophotometrically (see Table 2). In contrast, extracts obtained during massive flowering showed lower peak intensities for all major phenolic compounds, suggesting reduced accumulation of bioactive compounds at this stage. At the end of flowering, a partial recovery of identified phenolic compounds was observed, supporting the dynamic nature of secondary metabolite accumulation during plant development.

Although both UV-Vis and ED detectors were used for HPLC analysis, only UV-Vis chromatograms are presented because they provided clearer visualisation of the overall chromatographic profile and compound separation. The absolute values obtained by UV-Vis and ED detection differ because the detectors are based on different response mechanisms: UV-Vis detection depends on light absorption at the selected wavelength, whereas ED detection depends on electrochemical oxidation/reduction behaviour. Therefore, the two datasets are used to compare relative trends and detector-specific responses, not as interchangeable absolute concentrations. For this reason, both detector outputs were retained to show consistency of vegetation-stage trends across detection principles, but the values are interpreted as detector-specific semi-quantitative estimates rather than as directly comparable concentrations.

Semi-quantitative determination of identified phenolic compounds in *C. angustifolium* L. extracts obtained at different vegetation stages was performed using both detection methods, and the results are summarised in Table 3.

Semi-quantitative HPLC analyses revealed clear vegetation stage-dependent variations in the concentrations of identified phenolic compounds in *C. angustifolium* L. extracts (see Table 3). Oenothein B was the predominant compound across all stages, reaching its highest concentrations at the beginning of the flowering, as confirmed by both UV-Vis and electrochemical detection. Similar accumulation patterns were determined for 3,4-dihydroxybenzoic acid and ellagic acid, indicating maximal accumulation during early reproductive development. The lowest concentrations of all identified compounds were consistently detected during massive flowering, in agreement with reduced TPC, TFC and RSA values determined spectrophotometrically (see Table 2). Although the chromatographic profiles presented multiple additional peaks, only compounds confirmed by external standards were included in the semi-quantitative evaluation. The partial recovery of identified phenolic compounds observed at the end of flowering suggests dynamic redistribution of secondary metabolite resources during late developmental stages.

### 3.2. Influence of Extraction Method on Phytochemical Profiles in Selected Plant Samples

#### 3.2.1. Effect of Extraction Methods on TPC, TFC, and RSA

To evaluate the effectiveness of different extraction methods on the phytochemical properties of *C. angustifolium* L., *Q. robur* L. and *J. nigra* L. samples, TPC, TFC, and RSA were determined, as summarised in Table 4. The moisture content of room-dried samples ranged from 3.97% to 7.49%, confirming that the samples were properly dried and stored before extraction. Water content was taken into account to present the results on a dry-matter basis.

As shown in Table 4, the applied extraction strategy strongly affected the recovery of phenolic compounds and antioxidant capacity across all plant samples. Considerable differences in TPC, TFC, and RSA values were determined depending on solvent polarity, extraction method, and matrix-disrupting treatments, highlighting the importance of selecting the appropriate extraction method for reliable recovery of phytochemicals. When using solvents of different polarities for extraction, aqueous alcohol mixtures were most effective for the recovery of phenolic compounds. For all three plant species, 75% (*v*/*v*) methanol and 75% (*v*/*v*) ethanol in water solvents generally yielded the highest TPC, TFC, and RSA values, whereas pure methanol and water extracts yielded lower amounts. The highest TPC values were determined using 75% (*v*/*v*) methanol in water, reaching total polyphenolic compound content of 116.80 ± 3.29 mg RE/g in *C. angustifolium* L., 91.38 ± 3.18 mg RE/g in *Q. robur* L., and 126.46 ± 4.29 mg RE/g in *J. nigra* L. This indicates that aqueous alcohol solvent mixtures enhance extraction efficiency, most likely by improving the swelling of the plant matrix and better solubilization of compounds with varying polarities. Non-polar solvents (hexane, dichloromethane) and ethyl alcohol were not suitable for spectrophotometric evaluation under applied conditions. The formation of insoluble residues or suspensions of solid particles did not allow reliable determination of RSA, and the absorbance values of TPC and TFC were below the calibration range and therefore considered below the limit of quantification (LOD/LOQ values for TPC were 0.009/0.027 mg/mL, for TFC 0.006/0.017 mg/mL, and for RSA were 0.006/0.017 mg/mL). It should be noted that redissolution or solvent exchange could enable spectrometric analysis, but such modifications would alter the solubility profile of the extracted compounds and potentially change the qualitative and quantitative composition of the extracts. Therefore, to preserve the original extraction conditions and ensure solvent compatibility, no additional solvent adjustment was performed.

Ultrasound-assisted extraction using 75% (*v*/*v*) methanol in water further improved the recovery of phenolic compounds and antioxidant activity compared to a conventional 75% (*v*/*v*) methanol in water extraction. Compared with the corresponding conventional extraction, UAE increased TPC by 15.1%, TFC by 8.1%, and RSA by 16.3% in *C. angustifolium* L. extracts. In *Q. robur* L. extracts, the increases were 12.3% for TPC, 7.4% for TFC and 18.4% for RSA, while in *J. nigra* L. extracts, UAE increased TPC, TFC, and RSA by 14.1%, 8.6%, and 9.6%, respectively. This suggests that ultrasound treatment enhanced mass transfer and facilitated the release of phenolic compounds from the plant matrix. Hydrolysis-assisted extraction resulted in lower TPC, TFC, and RSA values than the corresponding 75% (*v*/*v*) methanol in water extraction. When comparing both hydrolyses, alkaline hydrolysis with 0.5 M NaOH produced higher TPC and RSA values, while acid hydrolysis with 0.5 M HCl generally resulted in a stronger decrease. Compared with conventional 75% (*v*/*v*) methanol extraction, alkaline hydrolysis decreased TPC by 12.1–18.3%, TFC by 43.5–52.6%, and RSA by 11.5–18.2%. Acid hydrolysis caused a greater decrease in TPC and RSA, reducing TPC by 30.9–38.4% and RSA by 31.9–39.2%, while TFC decreased by 18.4–21.6%. However, acid hydrolysis gave higher TFC values than alkaline hydrolysis, suggesting that acidic conditions may have promoted the release or conversion of flavonoids, whereas alkaline conditions may have caused partial degradation or transformation of flavonoids. Fermentation-assisted extraction using 75% (*v*/*v*) methanol after 48 h of lactic acid fermentation by *Lacticaseibacillus rahamnosus* GG produced modest, but statistically significant increases in TPC and RSA values compared to conventional 75% (*v*/*v*) methanol in water extraction. TPC increased by only 5.0–5.1%, and RSA by 5.0–5.6%. However, TFC values were statistically significantly lower after fermentation in all three plant materials, decreasing by 14.8–15.0%, indicating that short-term fermentation may promote the release of phenolic compounds, while some flavonoid compounds may be degraded or converted during fermentation.

In summary, the results demonstrate that extraction efficiency and antioxidant capacity are highly dependent on the extraction method and the solvent system. The most effective approach was ultrasound-assisted extraction with 75% (*v*/*v*) methanol in water, followed by aqueous alcohol solvents. Hydrolysis-assisted extraction produced lower values, particularly under acidic conditions, while fermentation-assisted extraction maintained or slightly improved TPC and RSA but reduced TFC. Overall, these findings confirm that the choice of extraction solvent and procedure should be aligned with the intended analytical or biological purpose.

#### 3.2.2. Comparative HPLC Profiling of Extracts Obtained by Different Extraction Methods

To further evaluate the influence of extraction methods on the qualitative and quantitative phenolic compound composition of the investigated plants, comparative HPLC-UV-Vis profiling was performed. Individual phenolic compounds were identified and quantified using the external standard method. In addition to quantifying the identified compounds, the total chromatographic peak area was calculated as the sum of all detected phenolic signals, serving as a semi-quantitative indicator of overall phenolic abundance. The results are summarised in Table 5.

Among the extraction methods based on solvent of different polarities, aqueous alcohol mixtures, particularly 75% (*v*/*v*) methanol in water and 75% (*v*/*v*) ethanol in water, yielded the most pronounced chromatographic responses across the plant samples investigated. Although only selected phenolic compounds were identified and quantified using available external standards, the total chromatographic peak area included all detected peaks, including unidentified compounds. Therefore, higher total peak area values do not necessarily correspond directly to higher concentrations of the individually quantified compounds. For example, in *C. angustifolium* L., the aqueous extract contained a higher concentration of oenothein B, whereas 75% (*v*/*v*) methanol in water extract yielded a higher peak area, indicating the extraction of a broader range of detected phenolic compounds. Thus, the total peak area should be interpreted as a semi-quantitative indicator of the overall chromatographic response, reflecting both identified and unidentified compounds. The HPLC results were generally consistent with spectrometric TPC, TFC, and RSA results presented in Table 4. Among the extraction methods, ultrasound-assisted extraction with 75% (*v*/*v*) methanol produced the highest overall chromatographic response, indicating the most efficient recovery of phenolic compounds under the applied conditions. This effect was reflected in the increased total peak area and higher concentrations of several quantified compounds, including oenothein B and 3,4-dihydroxybenzoic acid in *C. angustifolium* L., caffeic acid in *J. nigra* L., and gallic and ellagic acids in *Q. robur* L. plant extracts.

Hydrolysis-assisted extraction yielded lower overall chromatographic responses than aqueous alcohol 75% (*v*/*v*) methanol in water extraction. This decrease was observed for both alkaline and acid hydrolysis in all plant materials investigated. Among the different hydrolysis approaches, alkaline hydrolysis with 0.5 M NaOH generally resulted in higher total chromatographic peak areas than acid hydrolysis with 0.5 M HCl (22.9–34.4% higher, depending on the raw material). The decrease after acid hydrolysis was more pronounced, particularly in the total chromatographic peak area and in the concentrations of several quantified compounds. However, selected compounds showed different responses depending on the plant matrix, i.e., ellagic acid in *C. angustifolium* L. was higher after hydrolysis than after aqueous alcohol extraction. Fermentation-assisted extraction after 48 h of *Lacticaseibacillus rahamnosus* GG fermentation yielded chromatographic profiles that were generally comparable to those obtained by aqueous alcohol extraction. In all three plant materials, the total chromatographic peak areas were significantly higher than those of the corresponding conventional 75% (*v*/*v*) methanol in water extract (1.9% in *C. angustifolium* L., 7.9% in *J. nigra* L., and 6.5% in *Q. robur* L.), but lower than those obtained after ultrasound-assisted extraction. Changes in individual quantified compounds were compound and matrix-dependent, i.e., fermentation significantly decreased oenothein B in *C. angustifolium* L. by 5.3%, while 3,4-dihydroxybenzoic acid and ellagic acid increased by 7.5% and 57.1%, respectively. In *J. nigra* L., gallic acid, 3,4-dihydroxybenzoic acid, ferulic acid and trans-sinapic acid were slightly higher after fermentation (around 5.8–9.1%, depending on the compound), whereas caffeic acid decreased by 14.0%. In *Q. robur* L., gallic acid and ellagic acid increased by 25.0% and 27.8%, respectively, while rutin decreased by 16.8%.

Overall, the HPLC results confirm that the extraction method strongly influenced both the abundance and composition of phenolic compounds. While only a limited number of compounds were individually identified and quantified, the total chromatographic peak area captured a wider range of detected compounds and therefore provided complementary information to the targeted compound analysis. Among the tested approaches, UAE, with 75% (*v*/*v*) methanol in water extraction, showed the highest overall efficiency, whereas hydrolysis and fermentation mainly modified the phenolic profiles rather than uniformly increasing phenolic compound recovery.

### 3.3. Chemical Characterisation of SPE Fractions and Their Antiproliferative Activity

Building on our previous research results [11], the aim of this study was to determine which groups of phenolic compounds are primarily responsible for the determined antiproliferative activity of the crude plant extracts tested. To achieve this, the crude extracts with the strongest anticancer activity were extracted using solid-phase extraction with a C18 cartridge, yielding sequential fractions eluted with 0% (*v*/*v*) (F1), 30% (*v*/*v*) (F2), 60% (*v*/*v*) (F3), and 100% (*v*/*v*) (F4) methanol in water. Since methanol-containing extracts are not compatible with in vitro assays due to solvent-associated cytotoxicity [20], fractions F2 and F3 were subjected to solvent exchange and reconstituted in 5% (*v*/*v*) DMSO in water prior to biological evaluation. These fractions contained the highest concentrations of phenolic compounds and were therefore selected for further chemical characterisation and antiproliferative testing. The chemical composition of each SPE fraction was characterised by spectrophotometric assays and HPLC-UV-Vis and HPLC-ED analyses to confirm the composition of the fractions and to investigate the relationship between phytochemical profiles and biological activity. Statistical analyses included independent *t*-tests for fraction comparisons, repeated-measures ANOVA for time-dependent effects, and correlation analyses to assess relationships between chemical composition and antiproliferative activity.

#### 3.3.1. Phenolic Profile and Antioxidant Capacity of C18 SPE Fractions

The phytochemical composition and antioxidant capacity of the obtained C18 SPE fractions were evaluated using spectrophotometric assays (see Table 6).

As shown in Table 6, C18 SPE elution resulted in a clear redistribution of phenolic compounds across the aqueous wash and methanol-containing eluates in all investigated plant species. The initial 0% (*v*/*v*) methanol in water eluate represented the aqueous wash, containing unretained or weakly retained polar compounds, whereas the subsequent methanol-containing eluates reflected increasing elution strength. In general, the 30% (*v*/*v*) (F2) and 60% (*v*/*v*) (F3) methanol in water fractions exhibited substantially higher TPC and RSA values than aqueous wash and the 100% (*v*/*v*) methanol in water eluate, supporting their selection for further biological evaluation. Across all samples, no statistically significant differences were observed between F2 and F3 fractions in terms of total phenolic content, total peak area, or antioxidant activity (*p* > 0.05), indicating broadly comparable polyphenolic ant antioxidant potential. However, the total flavonoid content was significantly higher in the F3 fractions than in F2 (*p* = 0.045), suggesting selective enrichment of flavonoids in the stronger methanolic eluate.

The overall pattern was also reflected at the level of individual plant species, although some species-specific differences were observed. In the case of *C. angustifolium* L., the F2 fraction showed the highest TPC (66.85 ± 1.85 mg RE/g) and RSA (61.10 ± 2.44 mg RE/g) values, while the F3 had an increased flavonoid content (13.75 ± 0.21 mg RE/g), but comparatively lower antioxidant activity. When the solvent was exchanged to 5% (*v*/*v*) DMSO in water, the TPC and RSA contents in both fractions decreased slightly, although the separation ratio of the compounds did not change. A similar distribution was observed in *Q. robur* L., where F2 showed the highest TPC (43.22 ± 1.87 mg RE/g) and RSA (38.51 ± 1.48 mg RE/g), while F3 had the highest flavonoid content (12.62 ± 0.54 mg RE/g). The solvent exchange resulted in a slight decrease in the measured values without altering the overall fractionation profile. In *J. nigra* L., both F2 and F3 contained high levels of phenolic compounds, with F3 exhibiting the highest TPC (58.28 ± 1.97 mg RE/g) and RSA (51.20 ± 2.27 mg RE/g). After solvent exchange, F3 maintained relatively high TPC and RSA values (43.15 ± 1.56 and 41.19 ± 1.33 mg RE/g, respectively), indicating good stability of phenolic compounds during solvent removal and exchange. In the case of *J. regia* L., the total phenolic content was the lowest among the tested samples, resulting in TPC of 13.50 ± 0.48 mg RE/g and RSA of 11.24 ± 0.26 mg RE/g. In contrast to the other plant samples, *S. canadensis* L. showed the most pronounced fraction-dependent separation. The F3 fraction contained markedly higher TPC, TFC, and RSA values than the F2 fraction. These results indicate selective enrichment of flavonoid-rich and antioxidant-active compounds in the 60% (*v*/*v*) methanol in water fraction of *S. canadensis* L. Overall, the 30% (*v*/*v*) and 60% (*v*/*v*) methanol in water fractions generally showed comparable phenolic and antioxidant potential across most plant species, whereas *S. canadensis* L. represented a clear exception. Therefore, both fractions F2 and F3 were considered phytochemically relevant phenolic-rich fractions with distinct but biologically relevant profiles and were selected for further antiproliferative evaluation.

To further characterise the phenolic profiles of the obtained SPE fractions, HPLC UV-Vis and HPLC-ED analyses were performed for the 5% (*v*/*v*) DMSO in water fractions. The identified compounds, their quantified concentrations, and total chromatographic peak areas are presented in Table 7.

Chromatographic analysis confirmed that the intermediate-polarity SPE fractions (F2 and F3) were characterised by distinct and species-specific phenolic profiles. In general, F2 fractions were enriched in hydrolysable tannins and simple phenolic acids, whereas F3 fractions contained higher concentrations of flavonoid-type and cinnamic acid derivatives. The total chromatographic peak areas were consistent with spectrophotometric TPC and RSA results, confirming the enrichment of antioxidant-active phenolic compounds in these fractions. No statistically significant differences were observed between F2 and F3 fractions in total peak area (*p* > 0.05), indicating similar total phenolic abundance despite differences in composition. Therefore, the comparison between F2 and F3 was based not only on the low-abundance individually quantified compounds, but also on total chromatographic peak area, which includes unidentified peaks and represents the broader chromatographic profile of each fraction. Correlation analysis revealed a strong positive relationship between TPC, RSA and chromatographic peak areas (r = 0.716–0.973, *p* < 0.05), confirming that spectrometric assays reliably reflect overall phenolic content. In contrast, TFC showed weak or no correlation with antioxidant activity, suggesting that not all flavonoids contribute equally to radical scavenging capacity. These results confirm that C18 SPE fractionation effectively separates biologically active compounds into intermediate phenolic and flavonoid fractions, justifying their selection for subsequent antiproliferative evaluation.

#### 3.3.2. Antiproliferative Activity of SPE Fractions Against Cancer Cell Lines and HEK-293 Reference Cells

The antiproliferative activity of the selected SPE fractions was evaluated against five cancer cell lines, including 4T1 (mouse mammary carcinoma), A549 (human lung adenocarcinoma), Caki-1 (human kidney carcinoma), HCT116 (human colon carcinoma), and MCF7 (human breast adenocarcinoma). Anticancer activity was determined at different incubation periods (24, 48, and 72 h), and the half-maximal inhibitory concentration (IC_50_) values are summarised in Table 8.

The antiproliferative activity of the SPE fractions varied depending on plant species, SPE fraction, incubation time, and cancer cell line. IC_50_ values were calculated and expressed as mg dry raw material equivalent per mL (mg DRM/mL). Overall, IC_50_ values decreased with prolonged incubation, indicating increased antiproliferative activity with longer exposure. Linear mixed-model analysis of log_10_-transformed IC_50_ values confirmed a significant effect of incubation time in all tested cancer cell lines: 4T1 (F(2, 13.06) = 12.02, *p* = 0.001), A549 (F(2, 12.02) = 34.16, *p* < 0.001); Caki-1 (F(2, 15.01) = 9.626, *p* = 0.002), HCT116 (F(2, 11.04) = 17.44, *p* < 0.001), and MCF7 (F(2, 11.00) = 15.65, *p* < 0.001). Holm-adjusted post hoc comparisons showed significant differences between 24 h and 48 h, 24 h and 72 h, and 48 h and 72 h in all cell lines, confirming a consistent time-dependent decrease in IC_50_ values.

Although a universal main effect of fraction was not observed across the whole dataset, the plant species × fraction interaction was significant (*p* < 0.001), indicating that the relative antiproliferative activity of F2 and F3 depended on plant material. Therefore, plant-specific paired comparisons were performed using matched cell line-incubation time pairs. In *C. angustifolium*, F2 showed significantly lower IC_50_ values than F3, indicating stronger antiproliferative activity of the oenothein B-enriched fraction. In contrast, *Q. robur* L. showed significantly lower IC_50_ values for F3 than F2. A similar pattern was observed in *J. nigra* L. and *S. canadensis* L., where F3 also produced significantly lower IC_50_ values than F2. These findings indicate that, except for *C. angustifolium* L., the 60% (*v*/*v*) methanol in water fraction (F3) generally showed stronger antiproliferative activity than the 30% (*v*/*v*) methanol in water fraction (F2). In *J. regia* L., continuous paired comparison of IC_50_ values was limited because only Caki-1 cell line IC_50_ values were calculable for both F2 and F3. Therefore, the fraction-dependent response was evaluated mainly by the frequency of calculable IC_50_ values. The F3 fraction reached 50% inhibition in 12 of 15 tested conditions, whereas F2 reached 50% inhibition in only 3 of 15 conditions. McNemar’s exact test confirmed that IC_50_ values were calculated significantly more frequently for F3 than for F2 (*p* = 0.004), indicating stronger antiproliferative activity of the F3 fraction in *J. regia* L. within the tested concentration range. The strongest antiproliferative effects were generally observed after 72 h of incubation, with lower IC_50_ values indicating that a smaller amount of dry raw material equivalent was required to achieve 50% inhibition of cell viability. Particularly low IC_50_ values were observed for selected fractions of *C. angustifolium* L. (0.12 ± 0.005 mg DRM/mL against 4T1 cell line, F2 fraction) and *J. nigra* L. (0.29 ± 0.01 mg DRM/mL against HCT116 cell line; F3 fraction). Overall, these findings demonstrate that the antiproliferative activity of the selected fractions was not solely determined by total phenolic content or antioxidant activity, but also depended on plant species, fraction composition, cancer cell line, and incubation time.

Spearman correlation analysis was used to examine associations between chemical parameters and log_10_-transformed IC_50_ values (mg DRM/mL). Several unadjusted correlations indicated negative associations between total phenolic content or radical scavenging activity and IC_50_ values, suggesting that samples with higher phenolic content or radical scavenging activity tended to exhibit lower IC_50_ values and stronger antiproliferative activity. However, after Benjamini–Hochberg false discovery rate correction for multiple testing, none of the chemical parameter-IC_50_ correlations remained statistically significant. Therefore, these correlations should be interpreted as exploratory.

In addition to the associations between chemical parameters and IC_50_ values, correlation analysis also revealed positive relationships among several IC_50_ values obtained from different cancer cell lines and incubation times. This suggests that the relative anticancer activity response of the tested fractions was partly conserved across experimental models. Samples that showed comparatively lower IC_50_ values in one cell line or at one incubation time often showed lower IC_50_ values in other cell lines or at later time points as well. Such inter-cell-line and inter-time-point correlations indicate that some fractions may possess a broader anticancer activity profile rather than activity restricted to a single cancer cell. The strongest positive correlations were generally observed between IC_50_ values measured within the cell line across different incubation times, supporting a consistent time-dependent response pattern. Positive correlations between different cell lines further suggest that certain fractions induced comparable sensitivity patterns across multiple cancer cell lines. However, because these analyses involved multiple comparisons and were exploratory, the observed intercellular response correlations should be interpreted cautiously and confirmed in larger datasets. All tested SPE fractions exhibited minimal cytotoxicity towards the immortalised non-cancerous HEK-293 reference cell line, with cell viability remaining between 90% and 100% under the applied conditions.

## 4. Discussion

This study aimed to elucidate the relationship between phytochemical composition and antiproliferative activity of medicinal plant extracts using a structured, multi-level approach. The primary objective was to identify which groups of phenolic compounds contribute most to the anticancer activity of the crude extracts evaluated in our previous study [11] using C18 solid-phase extraction, i.e., the polarity of the compounds in the fraction, and to evaluate the resulting fractions in vitro. To support this objective, additional experiments were performed to evaluate how different extraction strategies affect phytochemical composition and investigate the vegetation stage-dependent variability in the accumulation of plant bioactive compounds in *C. angustifolium* L. samples. Together, these complementary methods allowed a comprehensive assessment of how extraction conditions, plant development, and fractionation together shaped the chemical profiles and biological potential of medicinal plant extracts.

### 4.1. Extraction Strategy and Phenolic Recovery

The comparison of extraction methods confirmed that aqueous alcohol mixtures were the most effective conventional solvents for the extraction of biologically active compounds, yielding the highest TPC, TFC, and RSA values among the solvent-only extracts. These differences were statistically significant (*p* < 0.05), confirming the key role of solvent polarity in the recovery of phenolic compounds. These results are in line with previous studies showing that water-alcohol mixtures improve the extraction of compounds with a wide range of polarities [32,33]. Ultrasound-assisted extraction using 75% (*v*/*v*) methanol in water further improved TPC, TFC, and RSA values compared to conventional 75% (*v*/*v*) methanol in water extraction, indicating that ultrasound treatment enhanced the release of biologically active compounds from the plant matrix, most likely through improved solvent penetration and mass transfer. Such results are consistent with previous reports showing that ultrasound-assisted extraction improves the recovery of phenolic compounds and flavonoids from plant matrices [34,35]. Hydrolysis-assisted extraction resulted in lower TPC, TFC, and RSA values than those obtained with 75% (*v*/*v*) methanol in water extraction. Acidic and alkaline hydrolysis may promote the release of soluble phenolic compounds from the plant matrix by breaking down bound, oligomeric, or polymeric structures, including tannin-related and lignan-related complexes. However, these reactions can occur simultaneously with degradation, oxidation, or structural transformation of already extractable phenolic compounds, and hydrolysis conditions have been shown to strongly influence phenolic compound recovery and antioxidant activity [36]. Under the conditions applied in this study, the decrease in biologically active compounds suggests that degradation or transformation processes predominated over the additional release of phenolic compounds. Under the conditions applied in this study, hydrolysis-assisted extraction did not improve the recovery of the measured phenolic and antioxidant-active compounds, suggesting that degradation, oxidation, or structural transformation may have outweighed the additional release of bound phenolic constituents. Therefore, future studies should optimise hydrolysis parameters to balance compound release and degradation. Fermentation-assisted extraction produced TPC and RSA values comparable to or slightly higher than those of conventional 75% (*v*/*v*) methanol in water extraction, while TFC decreased across all tested plant materials. Lactic acid bacteria fermentation has been reported to modify the phenolic composition and antioxidant activity in fruit matrices [37,38,39], probably through microbial or enzymatic transformations of the plant matrix and the partial degradation of complex phenolic structures into smaller, more soluble compounds. In the present study, maintained or slightly increased TPC and RSA values suggest that short-term fermentation may have promoted the release or transformation of some antioxidant-active phenolic compounds. However, the decrease in TFC indicates that certain flavonoids may have been degraded, metabolised, or converted into forms less detectable by the applied spectrometric method. Therefore, fermentation conditions should be further optimised, potentially separately for each plant species. Overall, these findings indicate that extraction efficiency and antioxidant capacity depend not only on solvent polarity but also on the balance between compound release and compound degradation or transformation during hydrolysis-assisted and fermentation-assisted extraction. Under the applied conditions, ultrasound-assisted extraction with 75% (*v*/*v*) methanol in water was the most effective approach, whereas hydrolysis-assisted extraction appeared to cause greater degradation or transformation of phenolic compounds than their additional release from the plant matrix. Therefore, further optimisation of hydrolysis and fermentation parameters is needed, potentially separately for each plant species.

### 4.2. Vegetation-Stage Effects in C. angustifolium L.

The influence of vegetation stage on the phytochemical composition was evident in the case of *C. angustifolium* L., where statistically significant differences were observed between development stages (*p* < 0.05). The beginning of flowering was characterised by the highest total phenolic, flavonoid and antioxidant content, as confirmed by distinct chromatographic profiles showing increased levels of major compounds, including oenothein B, ellagic acid and 3,4-dihydroxybenzoic acid. In contrast, samples collected during massive flowering were characterised by reduced peak intensity and lower spectrophotometric values, indicating a reduced accumulation of bioactive compounds at this stage. The partial recovery of phenolic compounds at the end of flowering indicates a dynamic regulation of secondary metabolite biosynthesis during plant development. These data are consistent with previous reports showing that phenolic compound accumulation is strongly influenced by vegetation stage, with early reproductive stages often associated with the highest biosynthetic activity [9]. Such variability highlights the importance of harvest time as a crucial factor in optimising the phytochemical yield and biological potential of medicinal plant materials. Although antiproliferative activity was not directly evaluated at different developmental stages, the observed differences in phenolic composition suggest that earlier developmental stages may yield extracts with greater biological potential.

### 4.3. C18 SPE Fractionation and Antiproliferative Activity

The application of C18 solid-phase extraction allowed for further separation of crude medicinal plant extracts into chemically distinct fractions, resulting in a clear redistribution of phenolic compounds along the polarity gradient. In all plant species studied, the F2 (30% (*v*/*v*) methanol in water) and F3 (60% (*v*/*v*) methanol in water) fractions contained the highest levels of phenolic compounds. Chemically, F2 was enriched in phenolic acids and hydrolysable tannins, while F3 contained higher proportions of flavonoids and cinnamic acid derivatives. This behaviour is consistent with the retention mechanisms of reversed-phase sorbents, where compounds of different polarity are eluted at increasing concentration of organic solvent [40]. Importantly, although solvent exchange reduced TPC and RSA, no significant changes in the relative distribution between fractions were observed, indicating the preservation of the phytochemical profile.

The antiproliferative results showed that the selected SPE fractions exhibited a clear time-dependent effect against cancer cell lines. Statistical analysis confirmed a significant effect of incubation time in all tested cancer cell lines, with IC_50_ values generally decreasing from 24 to 72 h. In addition, the significant plant species × fraction interaction indicated that the relative activity of F2 and F3 depended on plant material rather than following a single universal fraction effect. Plant-specific comparisons showed that F2 was more active in *C. angustifolium* L., whereas F3 showed stronger activity in *Q. robur* L., *J. nigra* L., and *S. canadensis* L. In *J. regia* L., this difference was mainly reflected in the higher frequency of calculable IC_50_ values for F3 than for F2. These findings indicate that antiproliferative activity depended not only on fraction type but also on plant species, incubation time, cancer cell line, and qualitative and quantitative phytochemical composition. Such observations are consistent with previous reports highlighting ellagic acid derivatives, gallic acid, flavonoids, and related phenolics as bioactive compounds capable of inducing apoptosis, modulating oxidative stress, and interfering with cancer cell signalling pathways [41,42,43].

The antiproliferative activity of the selected SPE fractions appeared to be associated with both qualitative and quantitative differences in their phytochemical composition. Although the 30% (*v*/*v*) and 60% (*v*/*v*) methanol in water fractions showed broadly comparable TPC and RSA values in several plant samples, their biological effects were not identical. In particular, the F2 fraction of *C. angustifolium* L. was characterised by oenothein B, whereas the F3 fraction contained ellagic acid and other phenolic compounds. Since both oenothein B and ellagic acid have been reported to exhibit anticancer activity [21,44], their distribution across different SPRE fractions may explain why both fractions showed biologically relevant antiproliferative effects. Therefore, the observed activity should not be attributed to a single dominant compound alone, but rather to the combined contribution of distinct phenolic profiles. The plant-dependent F2-F3 differences further suggest that fraction activity was shaped by the specific qualitative and quantitative composition of each plant extract. Nevertheless, the relatively low concentrations of individually quantified standard-confirmed compounds and the presence of unidentified chromatographic peaks limit direct attribution of the biological effects to specific compounds. Therefore, the observed antiproliferative activity should be interpreted at the fraction-profile level and may reflect combined effects of identified and unidentified constituents, including possible synergistic or antagonistic interactions within each fraction.

### 4.4. Correlation Analysis and Interpretation of Biological Activity

In line with the plant-dependent activity pattern, correlation analysis showed that the relationship between general phytochemical parameters (TPC, TFC) and antiproliferative activity was weak and inconsistent in most cell lines. Several unadjusted Spearman correlations indicated negative associations among TPC, RSA, and log_10_-transformed IC_50_ values, suggesting that samples with higher phenolic content or radical scavenging activity tended to exhibit lower IC_50_ values and stronger antiproliferative effects. However, after correction for multiple testing using the false discovery rate approach, none of these correlations remained statistically significant. Therefore, these relationships should be interpreted as exploratory trends rather than confirmed correlations. The lack of strong universal correlations suggest that the biological activity was likely influenced by specific compound, compound combinations, or cell-line dependent sensitivity rather than by total content of phenolic compounds or antioxidant activity alone. Differences in sensitivity between cancer cell lines were also observed, reflecting the variability of cellular responses to the tested fractions. Such variability may be related to differences in cellular metabolism, membrane permeability, and the molecular targets affected by phenolic compounds, including ellagic acid derivatives and flavonoids [45,46,47]. In HEK-293 immortalised non-cancerous reference cells, viability remained high under the applied conditions, indicating comparatively lower cytotoxicity in this reference model. However, this observation does not prove selective anticancer activity and should be verified in future work using tissue-matched normal cells, primary cultures, and selectivity-index calculations.

Based on correlation analysis, several cell lines exhibited similar response patterns to the SPE fractions tested. These correlations suggest that fractions showing relatively strong anticancer activity against one cancer cell line often tend to show strong activity against another. Positive correlations were particularly evident across several comparisons among the 4T1, A549, and Caki-1 cancer cell lines, indicating partially similar sensitivity patterns to the phytochemical profiles of the fractions. In contrast, HCT116 and MCF7 cells showed more variable and less consistent relationships with the other cell lines, suggesting possible differences in cellular sensitivity and underlying response mechanisms. These findings indicate that while some cancer cell models respond similarly to phenolic-containing extracts, others exhibit different responses, underscoring the importance of using multiple cell lines when evaluating anticancer potential. However, because these intercell-line correlations were exploratory and involved multiple comparisons, they should be interpreted cautiously.

The present results are consistent with our previous study [11], in which crude extracts rich in ellagic acid derivatives showed potent antiproliferative activity in multiple cancer cell lines. The current work extends these findings by showing that C18 SPE fractionation can further separate crude extracts into fractions with distinct phytochemical profiles and measurable antiproliferative activity. Importantly, the statistical analysis showed that the difference between F2 and F3 was plant-dependent. The oenothein B-enriched F2 fraction showed stronger activity in *C. angustifolium* L., whereas F3 showed stronger activity in *Q. robur* L., *J. nigra* L., *J. regia* L., and *S. canadensis* L. This indicates that the biological activity of SPE fractions cannot be explained by fraction polarity alone, but depends on the specific compound profile of each plant species. The activity retained by both F2 and F3 supports the hypothesis that different phenolic subclasses, including hydrolysable tannins, ellagic acid derivatives, flavonoids, and other medium-polarity compounds, may contribute to antiproliferative effects depending on plant species and cancer cell line. These findings support the hypothesis that the antiproliferative activity of medicinal plant extracts is likely driven by a combination of phenolic subclasses and possible synergistic interactions rather than by a single compound group alone [48]. Moreover, the lack of direct correlation between antioxidant capacity and anticancer activity is consistent with previous reports showing that extracts containing high levels of phytochemicals may exert biological effects through multiple mechanisms beyond antioxidant activity alone [49].

### 4.5. Study Limitations and Future Directions

In summary, this study demonstrates that combining extraction-method comparison, vegetation-stage profiling and C18 SPE fractionation can clarify how phenolic composition varies across plant material, processing conditions and biologically tested fractions. Several limitations should be acknowledged. First, rutin was used as a common reference standard for spectrometric and HPLC-based semi-quantification; this approach allows internal comparison across fractions but limits comparison with literature using gallic acid, Trolox, ascorbic acid or compound-specific calibration curves. Second, HPLC UV-Vis and HPLC-ED results are detector-dependent and should be interpreted as complementary semi-quantitative profiles rather than directly interchangeable absolute concentrations. Third, only standard-confirmed peaks were individually determined, while larger unidentified peaks may also contribute to biological activity. Fourth, HEK-293 cells are an immortalised non-cancerous reference model and do not replace primary cells or tissue-matched normal cell controls; therefore, conclusions about selective anticancer activity remain preliminary. Fifth, IC_50_ values expressed as mg DRM/mL are suitable for within-study comparisons of fractions but are not directly comparable with purified-compound IC_50_ values in the literature. Sixth, fermentation-assisted extraction was an exploratory workflow; pH dynamics, sterility controls, possible heat-related transformation during the short rotary-evaporation step, and mechanistic links to biological activity require further validation. Finally, the plant species differed in taxonomy, plant part and harvest stage, which supports assessment of plant-dependent responses but limits broad extrapolation. Future studies should focus on compound-specific calibration, identification of currently unidentified peaks, testing of isolated compounds and recombined fractions, and validation in primary or tissue-matched normal cell models.

## 5. Conclusions

This study shows that the extraction strategy and C18 SPE fractionation shape the phenolic profiles, antioxidant capacity and antiproliferative activity of selected medicinal plant extracts, while the single-species vegetation-stage analysis of *C. angustifolium* L. demonstrates the importance of harvest time for phenolic and antioxidant accumulation in this species. The main contribution of the work is not the confirmation that crude extracts are biologically active, which was shown previously, but the demonstration that fractionating previously active 75% (*v*/*v*) methanol in water extracts into phenolic-rich C18 SPE fractions reveals plant-dependent biological activity patterns. In particular, the activity of the selected fractions could not be attributed to a single universal fraction or to total phenolic content alone; rather, it depends on the qualitative and quantitative composition of each plant-derived fraction and on the cancer cell model tested. The extraction-method comparison and the vegetation-stage analysis should be interpreted as supportive workflows. They show that phenolic recovery and antioxidant capacity are strongly affected by extraction conditions and harvest stage, providing a chemical context for interpreting plant-dependent activity, but they do not constitute independent claims of universal extraction optimisation or direct prediction of biological activity. Overall, the results support the use of targeted fractionation as a useful approach for narrowing the relationship between phenolic composition and antiproliferative activity, while also indicating that extrapolation across different plant families, plant parts and phenological stages should be made cautiously. Future studies should focus on fewer plant materials, compound-specific quantification, and identification of currently unidentified peaks and validation using isolated or recombined compounds and additional normal-cell models.

## Figures and Tables

**Table 1:** Plant material collected and selected for the study.

No	Plant Name	Collection Date	Raw Material	Vegetation Stage
1	Fireweed (*C. angustifolium* L.)	30 May 2023	Aerial part (*Herba*)	Intensive growth
2		6 June 2023		Flower bud development
3		16 June 2023		Beginning of the flowering
4		20 June 2023		Massive flowering
5		26 June 2023		The end of the flowering
6	Pedunculate oak (*Q. robur* L.)	31 May 2023	Leaves	Leaf maturation
7	Black walnut (*J. nigra* L.)	6 October 2023	Pericarp	Ripening
8	Canadian goldenrod (*S. canadensis* L.)	25 July 2023	Aerial part (*Herba*)	Beginning of the flowering
9	Common walnut (*J. regia* L.)	30 September 2023	Pericarp	Ripening

**Table 2:** Vegetation stage-dependent variation in TPC, TFC, and RSA of *C. angustifolium* L.

Vegetation Stage	TPC *	TFC *	RSA *
Intensive growth	94.89 ± 3.71 ^b^	15.11 ± 0.52 ^b^	87.26 ± 3.49 ^b^
Flower bud development	90.49 ± 2.55 ^c^	14.42 ± 0.23 ^c^	82.88 ± 2.66 ^c^
Beginning of the flowering	116.8 ± 2.41 ^a^	20.29 ± 0.94 ^a^	101.49 ± 1.98 ^a^
Massive flowering	66.48 ± 1.93 ^e^	10.59 ± 0.41 ^e^	59.91 ± 2.37 ^e^
The end of the flowering	79.16 ± 1.86 ^d^	12.92 ± 0.33 ^d^	71.33 ± 2.19 ^d^

* TPC: total phenolic content; TFC: total flavonoid content; RSA: radical scavenging activity. Values are expressed as mg rutin equivalents (RE) per gram (mg RE/g). Values represent mean ± RSD (*n* = 3). Different superscript letters within the same column indicate statistically significant differences among vegetation stages according to Tukey’s HSD post hoc test (*p* < 0.05).

**Table 3:** Vegetation stage-dependent variation in identified phenolic compounds of *C. angustifolium* L. determined by HPLC UV-Vis and HPLC-ED analysis.

Vegetation Stage	Identified Compound	HPLC UV-Vis Analysis *	HPLC-ED Analysis *
Intensive growth	Oenothein B	1.14 ± 0.044	4.02 ± 0.141
	3,4-dihydroxybenzoic acid	0.39 ± 0.012	0.11 ± 0.002
	Ellagic acid	0.11 ± 0.003	0.18 ± 0.009
Flower bud development	Oenothein B	1.09 ± 0.031	4.06 ± 0.132
	3,4-dihydroxybenzoic acid	0.33 ± 0.012	0.16 ± 0.003
	Ellagic acid	0.14 ± 0.003	0.18 ± 0.009
Beginning of the flowering	Oenothein B	1.52 ± 0.059	5.14 ± 0.142
	3,4-dihydroxybenzoic acid	0.44 ± 0.012	0.21 ± 0.004
	Ellagic acid	0.16 ± 0.002	0.23 ± 0.008
Massive flowering	Oenothein B	0.88 ± 0.024	3.11 ± 0.127
	3,4-dihydroxybenzoic acid	0.14 ± 0.008	0.07 ± 0.001
	Ellagic acid	0.07 ± 0.001	0.19 ± 0.009
The end of the flowering	Oenothein B	1.04 ± 0.038	3.45 ± 0.102
	3,4-dihydroxybenzoic acid	0.18 ± 0.004	0.12 ± 0.002
	Ellagic acid	0.04 ± 0.001	0.14 ± 0.004

* HPLC UV-Vis and HPLC-ED values are expressed as mg rutin equivalents (RE) per gram (mg RE/g). Values represent mean ± RSD (*n* = 3). Values should be interpreted as detector-specific semi-quantitative estimates rather than as directly comparable absolute concentrations.

**Table 4:** Efficiency of extraction methods in terms of TPC, TFC, and RSA of *C. angustifolium* L., *Q. robur* L., and *J. nigra* L. extracts.

Extraction Method	Solvent	*C. angustifolium* L.			*Q. robur* L.			*J. nigra* L.		
		TPC *	TFC *	RSA *	TPC	TFC	RSA	TPC	TFC	RSA
Solvents of increased polarity	100% Hx	ND **	ND	NP ***	ND	ND	NP	ND	ND	NP
	100% DCM	ND	ND	NP	ND	ND	NP	ND	ND	NP
	100% ETAC	ND	ND	NP	ND	ND	NP	ND	ND	NP
	100% MeOH	79.05 ± 2.51	15.92 ± 0.48	62.97 ± 2.14	74.33 ± 2.11	14.01 ± 0.20	59.24 ± 1.88	96.39 ± 3.18	9.71 ± 0.29	83.74 ± 2.91
	100% H_2_O	65.66 ± 2.42	13.21 ± 0.42	52.51 ± 1.69	66.89 ± 2.33	13.12 ± 0.43	51.33 ± 1.96	85.55 ± 3.19	7.05 ± 0.10	78.22 ± 2.96
	75% (*v*/*v*) EtOH	111.67 ± 4.13	19.45 ± 0.76	95.16 ± 3.51	86.22 ± 1.22	17.30 ± 0.57	64.95 ± 2.19	122.08 ± 3.19	12.46 ± 0.29	96.74 ± 3.19
	75% (*v*/*v*) MeOH	116.80 ± 3.29	20.29 ± 0.79	101.49 ± 4.29	91.38 ± 3.18	18.97 ± 0.48	66.99 ± 3.01	126.46 ± 4.29	13.78 ± 0.22	107.97 ± 3.99
Ultrasound-assisted extraction	75% (*v*/*v*) MeOH	134.41 ± 4.10	21.93 ± 0.81	118.06 ± 3.28	102.63 ± 3.89	20.37 ± 0.74	79.34 ± 2.66	144.31 ± 5.17	14.96 ± 0.49	118.34 ± 3.71
Hydrolysis-assisted extraction	75% (*v*/*v*) MeOH + 0.5 M NaOH	102.72 ± 3.74	10.41 ± 0.22	89.82 ± 3.03	74.67 ± 2.12	8.99 ± 0.17	57.11 ± 2.44	106.37 ± 2.41	7.78 ± 0.22	88.31 ± 2.63
	75% (*v*/*v*) MeOH + 0.5 M HCl	71.99 ± 2.04	16.09 ± 0.46	62.22 ± 2.00	61.73 ± 2.01	14.88 ± 0.34	40.75 ± 1.11	87.33 ± 1.63	11.24 ± 0.44	73.51 ± 2.89
Fermentation-assisted extraction	75% (*v*/*v*) MeOH	122.64 ± 5.13	17.25 ± 0.76	106.56 ± 4.41	95.94 ± 3.66	16.12 ± 0.82	70.34 ± 2.33	132.89 ± 4.80	11.74 ± 0.34	113.96 ± 4.71

* TPC, TFC, and RSA values are expressed as mg rutin equivalents (RE) per gram (mg RE/g). Values represent mean ± RSD (*n* = 3); ** ND indicates that, for TPC and TFC assays, absorbance values were below the calibration range of the corresponding methods and were therefore considered below the limit of quantification; *** NP indicates that RSA was not performed due to the formation of insoluble residues in extracts obtained using non-polar solvents and ethyl acetate, which prevented reliable spectrophotometric measurements.

**Table 5:** Comparative HPLC UV-Vis profiling of phenolic compounds and total peak area in extracts obtained by different extraction methods.

Extraction Method	Solvent	Plant	Compound	HPLC UV-Vis C_RE_, mg/g *	Total Peak Area, mV/s **
Solvents of increased polarity	100% MeOH	*C. angustifolium* L.	Oenothein B	0.53 ± 0.012	9239.3 ± 275.4
			3,4-dihydroxybenzoic acid	0.11 ± 0.004	
			Ellagic acid	0.12 ± 0.002	
	100% H_2_O	*C. angustifolium* L.	Oenothein B	2.68 ± 0.091	29,949.2 ± 1226.8
			3,4-dihydroxybenzoic acid	0.41 ± 0.011	
			Ellagic acid	0.05 ± 0.001	
	75% (*v*/*v*) EtOH	*C. angustifolium* L.	Oenothein B	1.28 ± 0.043	25,886.0 ± 874.1
			3,4-dihydroxybenzoic acid	0.40 ± 0.012	
			Ellagic acid	0.09 ± 0.003	
	75% (*v*/*v*) MeOH	*C. angustifolium* L.	Oenothein B	1.52 ± 0.053	32,451.0 ± 1178.9
			3,4-dihydroxybenzoic acid	0.67 ± 0.020	
			Ellagic acid	0.07 ± 0.001	
	100% MeOH	*J. nigra* L.	Gallic acid	0.07 ± 0.002	8624.6 ± 241.9
			3,4-dihydroxybenzoic acid	0.04 ± 0.001	
			Caffeic acid	0.29 ± 0.013	
			Ferulic acid	0.09 ± 0.002	
			trans-sinapic acid	0.05 ± 0.001	
	100% H_2_O	*J. nigra* L.	Gallic acid	0.34 ± 0.012	15,449.3 ± 549.8
			3,4-dihydroxybenzoic acid	0.15 ± 0.004	
			Ferulic acid	0.17 ± 0.004	
			trans-sinapic acid	0.07 ± 0.002	
	75% (*v*/*v*) EtOH	*J. nigra* L.	Gallic acid	0.18 ± 0.003	18,354.6 ± 248.9
			3,4-dihydroxybenzoic acid	0.12 ± 0.004	
			Caffeic acid	0.41 ± 0.014	
			Ferulic acid	0.24 ± 0.009	
			trans-sinapic acid	0.22 ± 0.006	
	75% (*v*/*v*) MeOH	*J. nigra* L.	Gallic acid	0.17 ± 0.006	19,416.0 ± 739.4
			3,4-dihydroxybenzoic acid	0.12 ± 0.003	
			Caffeic acid	0.50 ± 0.001	
			Ferulic acid	0.24 ± 0.001	
			trans-sinapic acid	0.22 ± 0.008	
	100% MeOH	*Q. robur* L.	Gallic acid	0.04 ± 0.001	4930.8 ± 144.4
			Rutin	0.22 ± 0.01	
			Ellagic acid	0.07 ± 0.002	
	100% H_2_O	*Q. robur* L.	Gallic acid	0.08 ± 0.002	5247.8 ± 173.7
			Ellagic acid	0.05 ± 0.001	
	75% (*v*/*v*) EtOH	*Q. robur* L.	Gallic acid	0.07 ± 0.002	6592.5 ± 196.7
			Rutin	0.05 ± 0.001	
			Ellagic acid	0.18 ± 0.008	
	75% (*v*/*v*) MeOH	*Q. robur* L.	Gallic acid	0.06 ± 0.001	6900.6 ± 210.6
			Rutin	0.06 ± 0.002	
			Ellagic acid	0.18 ± 0.003	
Ultrasound-assisted extraction	75% (*v*/*v*) MeOH	*C. angustifolium* L.	Oenothein B	1.72 ± 0.064	38,172.0 ± 1221.0
			3,4-dihydroxybenzoic acid	0.74 ± 0.024	
			Ellagic acid	0.07 ± 0.001	
	75% (*v*/*v*) MeOH	*J. nigra* L.	Gallic acid	0.18 ± 0.008	22,059.0 ± 863.1
			3,4-dihydroxybenzoic acid	0.13 ± 0.004	
			Caffeic acid	0.61 ± 0.002	
			Ferulic acid	0.26 ± 0.011	
			trans-sinapic acid	0.24 ± 0.010	
	75% (*v*/*v*) MeOH	*Q. robur* L.	Gallic acid	0.08 ± 0.003	7819.3 ± 294.4
			Rutin	0.06 ± 0.001	
			Ellagic acid	0.19 ± 0.008	
Hydrolysis-assisted extraction	75% (*v/v*) MeOH + 0.5 M NaOH	*C. angustifolium* L.	Oenothein B	1.24 ± 0.039	25,489.1 ± 1169.9
			3,4-dihydroxybenzoic acid	0.58 ± 0.022	
			Ellagic acid	0.11 ± 0.004	
		*J. nigra* L.	Gallic acid	0.15 ± 0.004	16,967.8 ± 569.7
			3,4-dihydroxybenzoic acid	0.10 ± 0.003	
			Caffeic acid	0.36 ± 0.012	
			Ferulic acid	0.24 ± 0.010	
			trans-sinapic acid	0.22 ± 0.092	
		*Q. robur* L.	Gallic acid	0.05 ± 0.002	5764.4 ± 179.6
			Rutin	0.03 ± 0.001	
			Ellagic acid	0.15 ± 0.002	
	75% (*v*/*v*) MeOH + 0.5 M HCl	*C. angustifolium* L.	Oenothein B	0.64 ± 0.029	18,967.6 ± 832.6
			3,4-dihydroxybenzoic acid	0.41 ± 0.018	
			Ellagic acid	0.14 ± 0.003	
		*J. nigra* L.	Gallic acid	0.14 ± 0.006	13,796.5 ± 412.9
			3,4-dihydroxybenzoic acid	0.08 ± 0.003	
			Caffeic acid	0.32 ± 0.010	
			Ferulic acid	0.15 ± 0.006	
			trans-sinapic acid	0.14 ± 0.004	
		*Q. robur* L.	Gallic acid	0.06 ± 0.002	4469.9 ± 203.6
			Rutin	0.04 ± 0.002	
			Ellagic acid	0.10 ± 0.004	
Fermentation-assisted extraction	75% (*v*/*v*) MeOH	*C. angustifolium* L.	Oenothein B	1.44 ± 0.041	33,069.8 ± 1239.9
			3,4-dihydroxybenzoic acid	0.72 ± 0.032	
			Ellagic acid	0.11 ± 0.004	
		*J. nigra* L.	Gallic acid	0.18 ± 0.007	20,964.9 ± 898.9
			3,4-dihydroxybenzoic acid	0.13 ± 0.004	
			Caffeic acid	0.43 ± 0.018	
			Ferulic acid	0.26 ± 0.004	
			trans-sinapic acid	0.24 ± 0.008	
		*Q. robur* L.	Gallic acid	0.08 ± 0.002	7349.9 ± 234.6
			Rutin	0.05 ± 0.001	
			Ellagic acid	0.23 ± 0.008	

* Concentrations are expressed in rutin equivalents (RE) per gram (mg RE/g); ** Total peak area (mV/s) represents the summed chromatographic response of all detected phenolic peaks in the chromatogram and is not equivalent to the sum of individually quantified compounds. Only compounds confirmed using the external standard method were quantitatively determined; other detected peaks were included in the total peak area but not individually quantified. Values represent mean ± RSD (*n* = 3).

**Table 6:** TPC, TFC, and RSA in C18 SPE eluates and selected fractions before and after solvent exchange in *C. angustifolium* L., *Q. robur* L., *J. nigra* L., *J. regia* L., and *S. canadensis* L. samples.

Plant	SPE Eluate/Fraction	Spectrophotometric Analysis		
		TPC *	TFC *	RSA *
*C. angustifolium* L.	Aqueous wash, 0% (*v*/*v*) MeOH **	3.11 ± 0.10	1.19 ± 0.03	2.82 ± 0.08
	30% (*v*/*v*) MeOH (F2)	66.85 ± 1.85	4.19 ± 0.10	61.10 ± 2.44
	60% (*v*/*v*) MeOH (F3)	39.44 ± 1.16	13.75 ± 0.21	29.68 ± 0.97
	100% (*v*/*v*) MeOH (F4)	2.34 ± 0.08	1.52 ± 0.04	1.11 ± 0.03
	5% (*v*/*v*) DMSO (F2)	47.90 ± 2.11	3.15 ± 0.11	36.53 ± 1.48
	5% (*v*/*v*) DMSO (F3)	37.12 ± 1.56	10.33 ± 0.40	18.16 ± 0.75
*Q. robur* L.	Aqueous wash, 0% (*v*/*v*) MeOH)	ND ***	ND	ND
	30% (*v*/*v*) MeOH (F2)	43.22 ± 1.87	2.32 ± 0.08	38.51 ± 1.48
	60% (*v*/*v*) MeOH (F3)	39.18 ± 0.82	12.62 ± 0.54	22.33 ± 0.76
	100% (*v*/*v*) MeOH (F4)	5.53 ± 0.17	2.36 ± 0.07	3.18 ± 0.10
	5% (*v*/*v*) DMSO (F2)	38.03 ± 1.75	1.99 ± 0.03	35.37 ± 1.27
	5% (*v*/*v*) DMSO (F3)	25.97 ± 1.13	12.01 ± 0.42	21.45 ± 0.89
*J. nigra* L.	Aqueous wash, 0% (*v*/*v*) MeOH	3.58 ± 0.14	1.32 ± 0.05	2.21 ± 0.07
	30% (*v*/*v*) MeOH (F2)	55.34 ± 1.48	2.68 ± 0.11	45.11 ± 1.93
	60% (*v*/*v*) MeOH (F3)	58.28 ± 1.97	8.98 ± 0.21	51.20 ± 2.27
	100% (*v*/*v*) MeOH (F4)	3.22 ± 0.05	0.86 ± 0.02	2.35 ± 0.04
	5% (*v*/*v*) DMSO (F2)	39.49 ± 1.26	2.58 ± 0.10	37.24 ± 1.11
	5% (*v*/*v*) DMSO (F3)	43.15 ± 1.56	8.94 ± 0.32	41.19 ± 1.33
*J. regia* L.	Aqueous wash, 0% (*v*/*v*) MeOH	ND	ND	ND
	30% (*v*/*v*) MeOH (F2)	6.44 ± 0.28	1.10 ± 0.05	3.99 ± 0.14
	60% (*v*/*v*) MeOH (F3)	13.50 ± 0.48	3.93 ± 0.11	11.24 ± 0.26
	100% (*v*/*v*) MeOH (F4)	0.86 ± 0.03	0.56 ± 0.01	0.62 ± 0.01
	5% (*v*/*v*) DMSO (F2)	4.49 ± 0.15	1.04 ± 0.02	2.14 ± 0.08
	5% (*v*/*v*) DMSO (F3)	11.30 ± 0.48	3.20 ± 0.09	9.79 ± 0.25
*S. canadensis* L.	Aqueous wash, 0% (*v/v*) MeOH	2.98 ± 0.11	3.81 ± 0.08	2.39 ± 0.07
	30% (*v/v*) MeOH (F2)	23.23 ± 1.05	3.72 ± 0.11	12.04 ± 0.41
	60% (*v*/*v*) MeOH (F3)	37.21 ± 1.48	32.47 ± 1.31	27.83 ± 1.11
	100% (*v*/*v*) MeOH (F4)	7.96 ± 0.29	4.00 ± 0.11	6.88 ± 0.22
	5% (*v*/*v*) DMSO (F2)	17.38 ± 0.66	3.43 ± 0.10	12.35 ± 0.35
	5% (*v*/*v*) DMSO (F3)	38.76 ± 1.27	31.61 ± 1.08	26.01 ± 1.11

* Concentration expressed in rutin equivalents (RE) per gram (mg RE/g); ** The 0% MeOH eluate represented the aqueous wash and was not considered a separate SPE fraction for biological evaluation; *** ND represents values below the limit of quantification. Values represent mean ± RSD (*n* = 3).

**Table 7:** HPLC UV-Vis and HPLC-ED characterisation of phenolic compounds in C18 SPE fractions (F2 and F3) after solvent exchange to 5% (*v*/*v*) DMSO in water.

Plant	SPE Fraction	Compound	HPLC UV-Vis C_RE_, mg/g *	HPLC-ED C_RE_, mg/g *	Total Peak Area, mV/s **	Total Peak Area, µCoulombs ***
*C. angustifolium* L.	5% (*v*/*v*) DMSO (F2)	Oenothein B	0.58 ± 0.022	3.16 ± 0.141	11,326.85 ± 483.96	7424.02 ± 249.61
		3,4-dihydroxybenzoic acid	0.37 ± 0.011	0.08 ± 0.003		
	5% (*v*/*v*) DMSO (F3)	Ellagic acid	0.07 ± 0.003	0.09 ± 0.002	9938.96 ± 344.12	5566.51 ± 211.77
*Q. robur* L.	5% (*v*/*v*) DMSO (F2)	Gallic acid	0.03 ± 0.001	0.02 ± 0.001	1226.72 ± 43.33	697.76 ± 24.96
	5% (*v*/*v*) DMSO (F3)	Rutin	0.04 ± 0.002	0.01 ± 0.001	3948.96 ± 148.11	2447.56 ± 101.69
		Ellagic acid	0.16 ± 0.005	0.49 ± 0.022		
*J. nigra* L.	5% (*v*/*v*) DMSO (F2)	Gallic acid	0.04 ± 0.001	0.03 ± 0.001	3709.57 ± 96.88	2974.26 ± 116.96
		3,4-dihydroxybenzoic acid	0.09 ± 0.003	0.02 ± 0.001		
		Caffeic acid	0.31 ± 0.012	1.53 ± 0.066		
	5% (*v*/*v*) DMSO (F3)	Ferulic acid	0.10 ± 0.003	0.01 ± 0.001	5295.49 ± 163.78	3048.96 ± 124.63
		trans-sinapic acid	0.05 ± 0.002	0.11 ± 0.004		
*J. regia* L.	5% (*v*/*v*) DMSO (F2)	Gallic acid	0.03 ± 0.001	0.01 ± 0.001	979.04 ± 37.85	239.29 ± 8.93
		Chlorogenic acid	0.04 ± 0.002	0.02 ± 0.001		
	5% (*v*/*v*) DMSO (F3)	Ferulic acid	0.03 ± 0.001	0.01 ± 0.001	1330.94 ± 34.12	525.67 ± 22.82
		Rutin	0.03 ± 0.001	0.02 ± 0.001		
*S. canadensis* L.	5% (*v*/*v*) DMSO (F2)	Vanillic acid	0.10 ± 0.004	0.01 ± 0.001	693.13 ± 21.96	280.34 ± 11.85
	5% (*v*/*v*) DMSO (F3)	2-hydroxycinnamic acid	0.04 ± 0.002	0.02 ± 0.001	11,473.39 ± 481.48	4336.11 ± 148.88
		trans-cinnamic acid	0.44 ± 0.019	Not identified		

* Concentration expressed in rutin equivalents (RE) per gram (mg RE/g); ** Total peak area (mV/s) represents the summed chromatographic signal of all detected phenolic peaks; *** Total area of all registered peaks in µCoulombs obtained by HPLC-ED. Only compounds confirmed using the external standard method were quantitatively determined; other detected peaks were included in the total peak area but not individually quantified. Total peak area was therefore used as a broader chromatographic descriptor for statistical comparison with spectrophotometric parameters and antiproliferative activity. Values represent mean ± RSD (*n* = 3).

**Table 8:** Half-maximal inhibitory concentration (IC_50_) of SPE fraction against tested cancer cell lines, expressed in mg dry raw material equivalent per mL (mg DRM/mL; *n* = 3, RSD < 5%).

Plant	Fraction	Incubation Period	Half-Maximal Inhibitory Concentration (IC_50_), mg DRM/mL				
			4T1	A549	Caki-1	HCT116	MCF7
*C. angustifolium* L.	5% (*v*/*v*) DMSO (F2)	24 h	0.49 ± 0.01	1.43 ± 0.04	1.14 ± 0.03	1.76 ± 0.08	2.16 ± 0.09
		48 h	0.47 ± 0.02	0.86 ± 0.02	0.75 ± 0.02	1.00 ± 0.04	1.72 ± 0.07
		72 h	0.12 ± 0.005	0.79 ± 0.02	0.43 ± 0.02	0.58 ± 0.02	1.48 ± 0.05
	5% (*v*/*v*) DMSO (F3)	24 h	3.46 ± 0.11	3.24 ± 0.13	4.40 ± 0.17	6.47 ± 0.21	ND *
		48 h	1.47 ± 0.05	2.64 ± 0.11	3.06 ± 0.11	4.51 ± 0.22	ND
		72 h	1.25 ± 0.04	2.01 ± 0.07	3.22 ± 0.08	2.98 ± 0.09	8.76 ± 0.31
*Q. robur* L.	5% (*v*/*v*) DMSO (F2)	24 h	4.64 ± 0.18	5.77 ± 0.20	5.84 ± 0.22	ND	6.39 ± 0.18
		48 h	4.39 ± 0.18	4.34 ± 0.15	5.68 ± 0.21	13.61 ± 0.48	4.76 ± 0.11
		72 h	3.59 ± 0.14	4.07 ± 0.14	4.58 ± 0.08	10.45 ± 0.28	4.08 ± 0.14
	5% (*v*/*v*) DMSO (F3)	24 h	2.69 ± 0.09	ND	2.74 ± 0.09	9.94 ± 0.24	4.75 ± 0.13
		48 h	1.87 ± 0.04	4.85 ± 0.17	2.33 ± 0.07	6.70 ± 0.31	3.66 ± 0.11
		72 h	1.47 ± 0.05	3.34 ± 0.11	2.25 ± 0.08	6.39 ± 0.29	3.32 ± 0.08
*J. nigra* L.	5% (*v*/*v*) DMSO (F2)	24 h	2.50 ± 0.09	3.29 ± 0.10	2.61 ± 0.08	1.36 ± 0.04	5.36 ± 0.22
		48 h	1.15 ± 0.03	3.12 ± 0.08	1.68 ± 0.07	0.83 ± 0.03	3.38 ± 0.11
		72 h	1.13 ± 0.03	2.26 ± 0.09	1.78 ± 0.04	0.84 ± 0.02	2.76 ± 0.07
	5% (*v*/*v*) DMSO (F3)	24 h	0.79 ± 0.02	0.76 ± 0.02	0.39 ± 0.01	0.60 ± 0.02	0.62 ± 0.02
		48 h	0.60 ± 0.02	0.55 ± 0.01	0.42 ± 0.01	0.34 ± 0.01	0.54 ± 0.02
		72 h	0.58 ± 0.01	0.44 ± 0.02	0.39 ± 0.02	0.29 ± 0.01	0.41 ± 0.01
*J. regia* L.	5% (*v*/*v*) DMSO (F2)	24 h	ND	ND	0.49 ± 0.02	ND	ND
		48 h	ND	ND	0.37 ± 0.01	ND	ND
		72 h	ND	ND	0.29 ± 0.01	ND	ND
	5% (*v*/*v*) DMSO (F3)	24 h	ND	6.70 ± 0.22	2.84 ± 0.10	ND	4.10 ± 0.15
		48 h	2.97 ± 0.11	4.72 ± 0.18	1.61 ± 0.07	ND	4.04 ± 0.11
		72 h	2.13 ± 0.08	3.17 ± 0.11	1.19 ± 0.04	5.32 ± 0.21	3.94 ± 0.08
*S. canadensis* L.	5% (*v*/*v*) DMSO (F2)	24 h	8.69 ± 0.33	ND	9.33 ± 0.28	10.17 ± 0.34	9.13 ± 0.34
		48 h	7.42 ± 0.22	8.98 ± 0.40	7.03 ± 0.17	9.71 ± 0.18	8.75 ± 0.21
		72 h	5.12 ± 0.19	7.84 ± 0.21	6.01 ± 0.21	8.53 ± 0.33	7.74 ± 0.18
	5% (*v*/*v*) DMSO (F3)	24 h	3.67 ± 0.09	8.20 ± 0.33	ND	7.99 ± 0.21	5.87 ± 0.14
		48 h	3.39 ± 0.14	6.18 ± 0.21	6.10 ± 0.24	7.89 ± 0.28	5.42 ± 0.11
		72 h	1.25 ± 0.03	5.26 ± 0.17	3.51 ± 0.14	4.33 ± 0.14	4.91 ± 0.17

* ND—the half-maximal inhibitory concentration (IC_50_) was not determined because the used plant extract did not sufficiently inhibit (below 50%) cancer cell growth, and the regression curve could not be completed.

## Data Availability

The primary data summarised in this study are available on request from the corresponding author. The raw data are not publicly available because it is of little value when not analysed, compared, and summarised.

## Abbreviations

The following abbreviations are used in this manuscript:

ANOVA	Analysis of Variance
A549	Human Lung Adenocarcinoma
ATCC	American Type Culture Collection
Caki-1	Human Kidney Carcinoma
DCM	Dichloromethane
DMEM	Dulbecco’s Modified Eagle Medium
DPPH	2,2-diphenyl-1-picrylhydrazyl
DRM	Dry Raw Material
DVB/PMDS	Divinylbenzene/Polydimethylsiloxane
ED	Electrochemical Detection
EDTA	Ethylenediaminetetraacetic Acid
ETAC	Ethyl Acetate
EtOH	Ethanol
FBS	Fetal Bovine Serum
GC-MS	Gas Chromatography-Mass Spectrometry
HCT116	Human Colon Carcinoma
HEK-293	Human embryonic kidney
HPLC	High-Performance Liquid Chromatography
HSD	Honestly Significant Differences
Hx	Hexane
LOD	Limits of Detection
LOQ	Limits of Quantification
IC_50_	Half-maximal Inhibitory Concentration
MCF7	Human Breast Adenocarcinoma
MeOH	Methanol
MTS	3-(4,5-dimethylthiazol-2-yl)-5-(3-carboxymethoxyphenyl)-2-(4-sulfophenyl)-2H-tetrazolium
RE	Rutin Equivalents
RI	Retention Index
RPMI	Roswell Park Memorial Institute
RSA	Radical Scavenging Activity
RSD	Relative Standard Deviation
RT	Retention Time
SPE	Solid-Phase Extraction
TFA	Trifluoracetic Acid
TFC	Total Flavonoid Content
TPC	Total Phenolic Content
UV-Vis	Ultraviolet–Visible
UAE	Ultrasound-Assisted Extraction
4T1	Mouse Mammary Carcinoma
